# Origin, diversity, and biogeography of Antarctic scale worms (Polychaeta: Polynoidae): a wide‐scale barcoding approach

**DOI:** 10.1002/ece3.9093

**Published:** 2022-07-17

**Authors:** Dominique A. Cowart, Stefano Schiaparelli, Maria Chiara Alvaro, Matteo Cecchetto, Anne‐Sophie Le Port, Didier Jollivet, Stephane Hourdez

**Affiliations:** ^1^ Department of Evolution, Ecology, and Behavior University of Illinois at Urbana – Champaign Urbana Illinois USA; ^2^ Company for Open Ocean Observations and Logging (COOOL) La Réunion France; ^3^ Department of Earth, Environmental and Life Science (DiSTAV) University of Genoa Genoa Italy; ^4^ Italian National Antarctic Museum (MNA, Section of Genoa) University of Genoa Genoa Italy; ^5^ CNRS UMR 7144 ‘Adaptation et Diversité en Milieux Marins’ (AD2M) Team ‘Dynamique de la Diversité Marine’ (DyDiv), Station Biologique de Roscoff Sorbonne Université Roscoff France; ^6^ Laboratoire d'Ecogéochimie des Environnements Benthiques (LECOB), Observatoire Océanologique de Banyuls UMR 8222 CNRS‐Sorbonne Université Banyuls‐sur‐mer France

**Keywords:** Antarctic biogeography, benthic invertebrate, DNA barcoding, gene flow, polynoid, Southern Ocean, species connectivity

## Abstract

The Antarctic marine environment hosts diversified and highly endemic benthos owing to its unique geologic and climatic history. Current warming trends have increased the urgency of understanding Antarctic species history to predict how environmental changes will impact ecosystem functioning. Antarctic benthic lineages have traditionally been examined under three hypotheses: (1) high endemism and local radiation, (2) emergence of deep‐sea taxa through thermohaline circulation, and (3) species migrations across the Polar Front. In this study, we investigated which hypotheses best describe benthic invertebrate origins by examining Antarctic scale worms (Polynoidae). We amassed 691 polynoid sequences from the Southern Ocean and neighboring areas: the Kerguelen and Tierra del Fuego (South America) archipelagos, the Indian Ocean, and waters around New Zealand. We performed phylogenetic reconstructions to identify lineages across geographic regions, aided by mitochondrial markers cytochrome c oxidase subunit I (Cox1) and 16S ribosomal RNA (16S). Additionally, we produced haplotype networks at the species scale to examine genetic diversity, biogeographic separations, and past demography. The Cox1 dataset provided the most illuminating insights into the evolution of polynoids, with a total of 36 lineages identified. *Eunoe* sp. was present at Tierra del Fuego and Kerguelen, in favor of the latter acting as a migration crossroads. *Harmothoe fuligineum*, widespread around the Antarctic continent, was also present but isolated at Kerguelen, possibly resulting from historical freeze–thaw cycles. The genus *Polyeunoa* appears to have diversified prior to colonizing the continent, leading to the co‐occurrence of at least three cryptic species around the Southern and Indian Oceans. Analyses identified that nearly all populations are presently expanding following a bottleneck event, possibly caused by habitat reduction from the last glacial episodes. Findings support multiple origins for contemporary Antarctic polynoids, and some species investigated here provide information on ancestral scenarios of (re)colonization. First, it is apparent that species collected from the Antarctic continent are endemic, as the absence of closely related species in the Kerguelen and Tierra del Fuego datasets for most lineages argues in favor of Hypothesis 1 of local origin. Next, *Eunoe* sp. and *H. fuligineum*, however, support the possibility of Kerguelen and other sub‐Antarctic islands acting as a crossroads for larvae of some species, in support of Hypothesis 3. Finally, the genus *Polyeunoa*, conversely, is found at depths greater than 150 m and may have a deep origin, in line with Hypothesis 2. These “non endemic” groups, nevertheless, have a distribution that is either north or south of the Antarctic Polar Front, indicating that there is still a barrier to dispersal, even in the deep sea.

## Graphical abstract

We used scale worms as a representative group for Antarctic marine benthic animals to better understand their evolutionary history, and possibly predict how  they could respond to warming temperatures. Using genetic barcode data from over 600 scale worms, we were able to comprehend the history of this group during past warming and cooling geological periods. These events had profound effects on the genetic diversity of scale worms that are still detectable in present populations. For one, the geographic separation made by the Antarctic Circumpolar Current identified that northern animals are unlikely to colonize waters off of the Antarctic continent, raising concerns for the future of colder biological communities in times of warming.

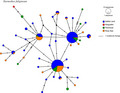

## 1 INTRODUCTION

Marine benthic biodiversity of the Southern Ocean has been significantly influenced by the unique geologic and climatic history of Antarctica (Aronson & Blake, 2001; Clarke & Crame, 1989; Convey et al., 2009; Majewski et al., 2021). The final separation of Antarctica from other Gondwana fragments coincided with the opening of the Tasmanian and Drake Passages, between 29 and 23 mya, allowing the establishment of the clockwise flowing Antarctic Circumpolar Current (ACC) (Lawver & Gahagan, 2003; Pfuhl & McCave, 2005). Since then, several glaciation periods triggered cycles of ice cap formation and retreat on 40,000‐ to 100,000‐year base cycles, shaping the Antarctic terrestrial and marine biota to the extent we see today (Kemp et al., 2010; Verducci et al., 2009). The Antarctic Polar Front (APF), a powerful jet of ACC that flows eastward around Antarctica, is known to create a geographic and thermal barrier to north–south faunal dispersal, thus maintaining the general isolation of Antarctic marine biota (Clarke et al., 2005; Verheye et al., 2017). As a result of these restrictive environmental conditions, unusually high levels of endemism, eurybathy, and stenothermal tolerance have been observed in many Antarctic taxa (Brey et al., 1996; Peck, 2002), accompanied by adaptive radiation and speciation events that led to high diversity levels in some groups (Bowen et al., 2020; Chenuil et al., 2018; Convey et al., 2014; Fassio et al., 2019; Peck, 2018).

Evolutionary origins of Antarctic marine benthos, and especially how they relate to the above‐mentioned geological and historical processes, have frequently been investigated in the context of three major hypotheses. These hypotheses, originally detailed in Knox & Lowry, 1977, suggest that contemporary species might be the descendants of (1) temperate Gondwana fauna whom evolved and adapted in situ to cold conditions (Antarctic origin, “species flock” theory, Lecointre et al., 2013); (2) cold‐adapted deep‐sea groups that emerged onto shallow systems (deep‐sea origin, polar emergence, Strugnell et al., 2011); (3) recently derived colonizers from South America and elsewhere, using sub‐Antarctic islands as stepping stones (external origin, Poulin et al., 2014). Determining which of these hypotheses best fits for the origin of Antarctic benthos has been further complicated by the consideration of bidirectional exchange of fauna between the shallow Antarctic shelves and the deep sea, driven by thermohaline circulation differential pulses and flow directions that characterize glacial and interglacial cycles (Clarke, 2008; Díaz et al., 2011; Strugnell et al., 2008, 2011). Analyses must also consider the dynamics of ice extension and retraction over shelf areas, which may influence habitat and refugia availability, as well as the difficulties in reconstructing evolutionary histories of groups lacking fossil records (Clarke & Crame, 1992; Gutt, 2001; Lau et al., 2020; Rogers, 2007).

Modern advancements in molecular techniques have provided a new perspective in the study of biogeographical events (Clarke, 2008). Molecular studies based upon either mitochondrial genes only or both mitochondrial and nuclear genes have allowed a powerful assessment of current species diversity, phylogeographic information, and past demography. For example, the use of both mitochondrial and nuclear genes confirmed the isolation and adaptive radiation of some Antarctic benthic taxa, including amphipods of the genus *Epimeria* Costa in Hope, 1851 (Verheye et al., 2017), littorinid, and velutinid gastropods (Fassio et al., 2019; Williams et al., 2003), while mitochondrial gene investigations identified the Notothenioidei fishes' species flock (Near, 2004; Near et al., 2004), all of which support the Antarctic origin hypothesis. Additional studies have identified bidirectional exchanges of lineages between the shallow shelves and deep sea over evolutionary time (Brandt et al., 2007, nuclear), which may complicate the generally adopted unidirectional colonization of fauna from deep to the shallow (i.e., the deep‐sea origin hypothesis, “polar emergence” vs. “polar submergence”; Díaz et al., 2011; Strugnell et al., 2011, mitochondrial). This has most notably been shown by the genetic similarity of closely related taxa living across large depth ranges, including the Foraminifera genus *Epistominella* (Husezima & Maruhasi, 1944; Pawlowski et al., 2007, nuclear, submergence) and the octopod genus *Benthoctopus* Grimpe, 1921 (now a synonym of *Bathypolypus* Grimpe, 1921) (Strugnell et al., 2008, 2011, submergence/emergence), or by the fossil shell morphology of deep‐sea pectinid bivalves (Berkman et al., 2004) and muricid gastropods (Barco et al., 2012).

Despite the isolation of Antarctic biota, the ACC has been found not to be an absolute barrier to exchanges (Barnes et al., 2006). For example, there is faunal overlap between South America and Antarctic peninsula regions, particularly within the Scotia Arc (Moore et al., 2018) and molecular and mathematical tools identify support for faunal movement across the APF (Leese et al., 2010). For example, a recent larval dispersal model suggests that the crossing of the APF, from the Antarctic continent toward sub‐Antarctic archipelagos such as Kerguelen, is possible (Brasier et al., 2017; González‐Wevar et al., 2021). Transport, nevertheless, is likely rare, as few benthic species are found on both sides of the Southern Ocean (Antezana, 1999; Clarke et al., 2005). Taxa most likely to cross the APF must have a high dispersal potential in the form of pelagic stages that are easily transported by currents (Alve & Goldstein, 2003; Leese et al., 2010), and some species could disperse below the APF or be transported by floating debris (“rafting,” Highsmith, 1985; Helmuth et al., 1994; Waters, 2008; González‐Wevar et al., 2021). The presence of closely related taxa on either side of the ACC is more common, with species divergence times younger than the continental separation and subsequent formation of the APF (Poulin et al., 2014). This has been indicated by the co‐occurrence of sister species in the sub‐Antarctic islands for the Antarctic bivalves of the genus *Limatula* (Wood, 1839; Page & Linse, 2002), the brittle star *Astrotoma agassizii* (Lyman, 1875; Hunter & Halanych, 2008), the limpet genus *Nacella* (Schumacher, 1817; González‐Wevar et al., 2010, 2011), and the crinoid *Promachocrinus kerguelensis* (Carpenter, 1879; Hemery et al., 2012; Wilson et al., 2007).

The establishment of glaciation cycles, driving ice sheet extension and iceberg scour across benthic habitats, also promoted speciation, and acted as a “biodiversity pump” (Clarke & Crame, 1989, 1992). The advancing ice limited suitable habitat along the continental shelf, thus fragmenting species distributional ranges and constraining food availability (Clarke & Crame, 2010). To survive, fauna was postulated to have migrated down the continental slope, into the deep sea, or sought refuge within ice‐free locations at shallower depths (Thatje et al., 2005, 2008). These ice movements isolated populations and forced genetic and ecological divergence as taxa adapted to new conditions, creating geographically widespread lineages such as the crinoid *P. kerguelensis* (Hemery et al., 2012) and the limpets of the genus *Nacella* (González‐Wevar et al., 2010).

**FIGURE 1:**
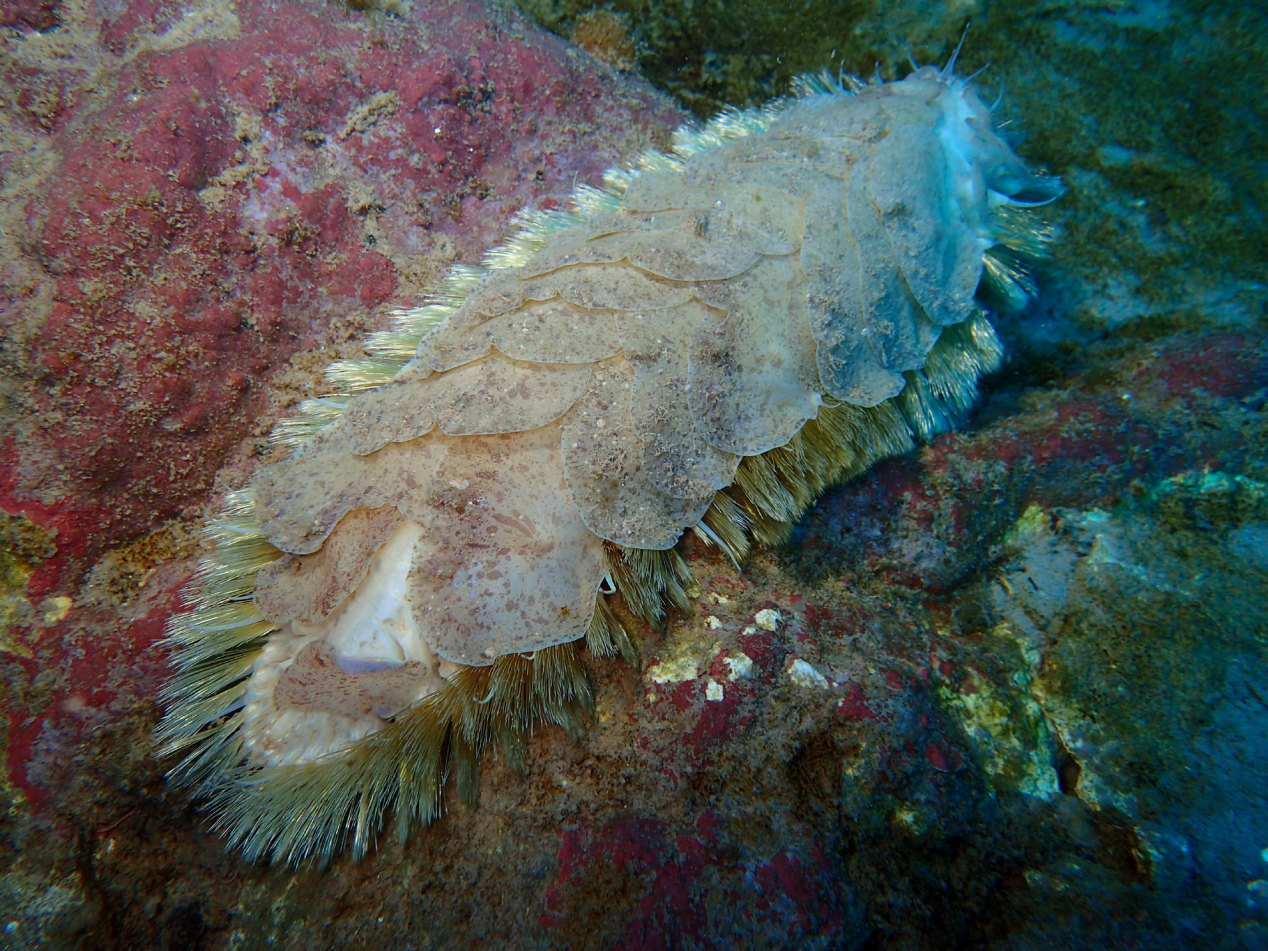
An image of the species *Eulagisca uschakovi* (Annelida, Polynoidae), near the Dumont d'Urville Station, Antarctica, taken at 30 m. The individual's approximate length is 25 cm. Copyright Pierre Chevaldonné

Polychaetes, totalling over 500 currently accepted species in the Register of Antarctic Marine Species (RAMS) database (De Broyer et al., 2022) occurring in the Southern Ocean, represent an important component of Antarctic benthic community both in terms of species richness and energy flow (Aronson et al., 2007; Clarke & Johnston, 2003; Gambi et al., 1997). Within this group is the family Polynoidae (“polynoids”), commonly known as “scale worms,” which includes over 50 known species living in the Southern Ocean (De Broyer et al., 2022, see Figure 1). Polynoids are predators that feed on small arthropods and mollusks and thus have an important role in the benthic food web (Jumars et al., 2015). These attributes, in addition to a variety of life history strategies, a high species diversity and abundance, and a widespread geographic and bathymetric distribution in the Southern Ocean (Clarke & Johnston, 2003; Gambi et al., 2001; Jumars et al., 2015), identify polynoids as an ideal candidate for providing additional insights into the evolutionary origins of Antarctic marine benthos.

We provide an exposition of historical relationships of Antarctic, sub‐Antarctic, South American, and New Zealand members of Polynoidae through assembled phylogenetic analyses of mitochondrial gene fragments of barcoded specimens. Mitochondrial genes, as well as nuclear genes, are often employed during evolutionary reconstructions; mitochondrial genes are frequently used given their relatively fast mutational rates, usefulness for detecting potential genetic breaks, and the wealth of sequence data already present in public repositories, just to name a few advantages of this marker type (Hebert et al., 2003; Roe & Sperling, 2007). Additionally, mitochondrial cytochrome c oxidase subunit I (Cox1) has been preferred for molecular demographic investigations, as it has higher intraspecific sequence variance (*see* Soler‐Membrives et al., 2017). Here, we detail an expansive Antarctic polynoid dataset to improve understanding of the evolutionary history of polynoids as another puzzle piece of Antarctic species origins, to further illuminate what combination of the three origins hypothesis is responsible for overall benthic speciation in the Antarctic.

## 2 MATERIALS AND METHODS

### 2.1 Study areas and sample collection

**FIGURE 2:**
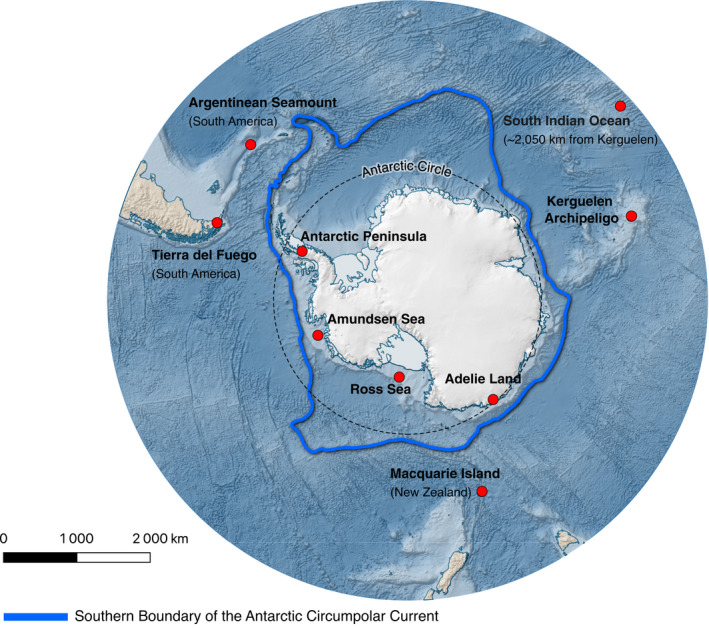
Locations from where polynoid individuals were collected that also includes locations for taxa whose sequences were previously published and used in this study (*see* Table A2). The center of minimum distance between the South Indian Ocean sites (*n* = 4, Serpetti et al., 2017) was calculated (http://www.geomidpoint.com/); this distance was identified as being located approximately 2050 km north of the Kerguelen archipelago, for reference. This map was produced with the aid of QGIS and the Quantarctica3 mapping environment (Matsuoka et al., 2021)

Polynoids were retrieved from three regions along the Antarctic continent: Adélie Land, Ross Sea and Antarctic Peninsula. Additional specimens were also obtained from the sub‐Antarctic Kerguelen and the Tierra del Fuego (Chile, South America) archipelagos and New Zealand (Figure 2). Specimen collections were performed across several years aboard multiple research vessels, aided by an assortment of sampling equipment (Summary A1). Sampling efforts employed trawls, dredges, and grabs, among other methods, and various sampling designs were implemented to answer specific research questions. These efforts allowed considerable taxon sampling, providing a total of 691 sequences overall that include previously published sequences. Specimens that were processed within the context of this study were preserved either in 96° ethanol or at −80°C. Prior to further analyses, each collected individual was identified to the lowest possible taxonomic level based on morphological characters. Specific sampling permissions were obtained within the framework of each of the different expeditions (*see* Summary A1).

### 2.2 DNA extraction, amplification, and specimens barcoding

Genomic DNA of polynoids retrieved from Antarctic regions, sub‐Antarctic Kerguelen, and the Tierra del Fuego archipelagos, apart from most individuals collected from the Ross Sea, was extracted by clipping several parapodia from each individual and following a modified CTAB protocol (Doyle & Doyle, 1987). Parapodia were placed in 1.5‐ml tubes filled with 500 μl of CTAB buffer solution, 5 μl of 10 mg/ml proteinase K, and 2 μl of ß‐mercaptoethanol. Tubes were inverted prior to incubation at 55°C for at least 3 h until tissue was digested. After the digestion step, tubes were centrifuged for 5 min at 11,000 rpm, after which the supernatant was transferred to a clean 1.5‐ml tube containing 400 μl of chloroform‐isoamyl alcohol 24:1. Following inversion for 2 min, tubes were centrifuged for 10 min at 4°C and 11,000 rpm. The resulting top aqueous layer was transferred to a clean 1.5‐ml tube containing 250 μl of isopropanol for precipitation at −20°C for 1 h. The DNA pellet was then precipitated by centrifuging tubes at 11,000 rpm for 10 min, and the supernatant was eliminated. Pellets were washed twice by adding 500 μl of 70% ethanol to each tube prior to centrifugation at 11,000 rpm for 10 min. Finally, pellets were air‐dried until no visible liquid remained and then were resuspended in a maximum of 150 μl of 0.5X TE buffer and stored at −20°C prior to end‐point PCR amplification.

PCR assays were performed using extracted DNA to amplify fragments of two mitochondrial genes, cytochrome c oxidase subunit I (Cox1) and 16S ribosomal RNA (16S), using previously developed primers (Table A1). PCR master mixes were prepared for every individual sample as follows: 18.5 μl of PCR‐grade water, 2.5 μl of 10X buffer, 1 μl of 50 mM MgCl_2_, 0.5 μl of 2.5 mM dNTP, 0.1 μl of UptiTherm DNA polymerase (Interchim), 0.25 μl of each primer (10 mM), and 1 μl of DNA extract from a single individual. All reactions were run on a GeneAmp® PCR System 9700 thermocycler (Applied Biosystems) under the following conditions: an initial cycle of 95°C for 5 min, 52°C for 45 sec, and 72°C for 4 min; 35 cycles of 95°C for 40 sec, 52°C for 45 sec, 72°C for 45 sec, followed by a final extension at 72°C for 7 min. Resulting PCR products were electrophoresed on 1% agarose gel stained with GelRed™ (Biotium) to visualize DNA quality and size prior to submission to Eurofins Scientific for purification and Sanger sequencing in both directions, using ABI BigDye® Terminator v3.1 Cycle sequencing kit (Applied Biosystems).

Most of the individuals sampled from the Ross Sea were processed at the Canadian Center for DNA Barcoding (CCDB—University of Guelph, Canada). Briefly, a portion of an elytron corresponding to approximately 1 mm was taken from each individual and placed in an 8 × 12 well microplate filled with absolute ethanol. The microplate was then shipped to the University of Guelph and processed following the CCDB automated standard protocols for extraction, amplification using primers in Table A1, and sequencing (http://ccdb.ca/resources/).

### 2.3 Species diversity and statistical analyses

Resulting sequence chromatograms were visualized, assembled, and edited using Codoncode Aligner 7.1.2 (CodonCode Corporation) and Geneious v.10.0.5 (Kearse et al., 2012). Putative sequence identities were assessed by matching each sequence against all available Cox1 and 16S sequence data within the National Center for Biotechnology Information (NCBI) GenBank public database, implementing the blastn algorithm under default parameters (Johnson et al., 2008). Downstream analyses were performed on the Cox1 dataset. To aid in the phylogenetic reconstructions, the determination of species geographic range and diversity at each location, additional polynoid Cox1 sequences were obtained from GenBank, including those originating from individuals taken from the Amundsen Sea continental region (*n* = 12, Brasier et al., 2017). For *Polyeunoa laevis* (McIntosh, 1885) specifically, additional sequences were obtained from the Amundsen Sea (*n* = 43, Bogantes et al., 2020), the Ross Sea (*n* = 2, Gallego et al., 2014; *n* = 21, Bogantes et al., 2020), the Peninsula (*n* = 45, Bogantes et al., 2020), the Indian Ocean (*n* = 4, Serpetti et al., 2017), Argentinean seamounts (*n* = 5, Bogantes et al., 2020), and New Zealand Macquarie Ridge (*n* = 7, Schiaparelli unpublished) (*see* Table A2). Sequence alignments were performed with the aid of Seaview v.4 (Gouy et al., 2010). Phylogenetic trees were generated with PhyML using a GTR I + Γ model of substitutions, with an optimization of the gamma shape of the mutation rate and proportion of invariant sites, as suggested by jModelTest (Posada, 2008). This approach allowed the determination of sequence clusters assumed to correspond to species and assigned specimens to these clusters. To determine whether the sampling and sequencing effort were extensive enough to uncover all the species diversity present, accumulation curves based on the sequence information were obtained by implementing the function *specaccum* within the R “vegan” package (version 2.5–2, Oksanen et al., 2016). For this approach, samples from targeted studies on *P. laevis* were omitted to avoid bias.

Further investigation of distribution patterns was performed by constructing Cox1 haplotypes networks for five species with >30 individual sequences assigned: *Barrukia cristata* (Willey, 1902), *Harmothoe crosetensis* (McIntosh, 1885), *Harmothoe fuligineum* (Baird, 1865), *Harmothoe magellanica* (McIntosh, 1885), and *P. laevis*. Networks were generated using Phylip alignments imported into PopART (Leigh & Bryant, 2015) and run using the Templeton, Crandall and Sing (TCS, *see* Templeton et al., 1992) network option under default parameters (Clement et al., 2002). The absence of barriers to dispersal between populations within a species was tested in DnaSP based on Fst values for the different populations (version 4.0, Rozas et al., 2003). In addition to calculating haplotype and nucleotide diversities, testing the hypothesis of haphazard deviation from neutral evolution and potential population expansion was performed by generating Tajima's D (Tajima, 1989) and Fu and Li's statistics (Fu, 1997) using DnaSP. Population expansion was further tested by modeling the frequency of pairwise differences for large populations with constant size, and for an expanding population in DnaSP (population size changes module). The goodness of fit with the observed pairwise differences was tested with a χ^2^ test. As there is no mutation rate published for polynoids, the age of the bottleneck (T = Tau/2 μ) was calculated with a more generic mutation rate of 1.8% for Cox1 (Knowlton, 1993) and assuming one generation per year.

## 3 RESULTS

**FIGURE 3:**
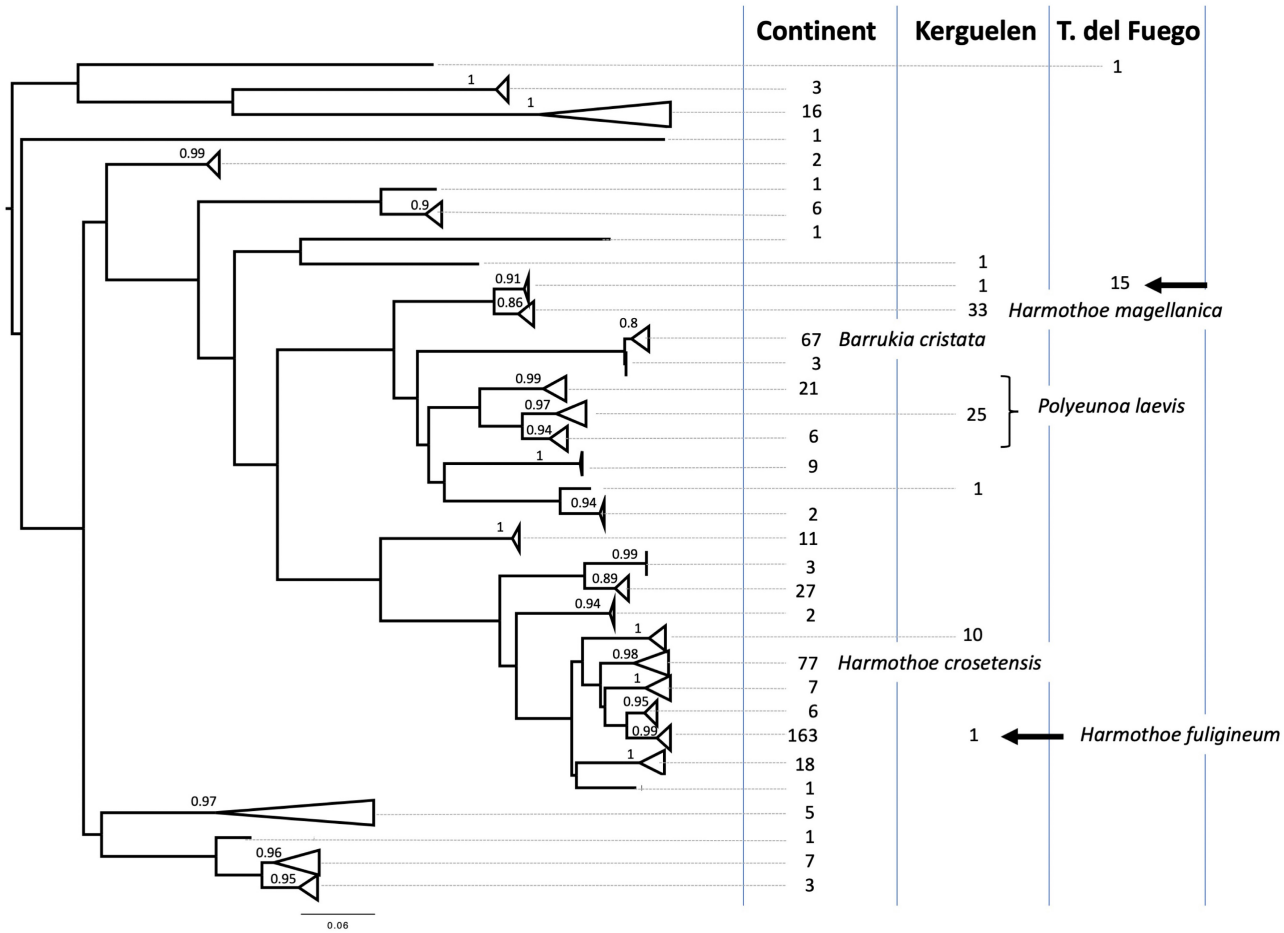
Mitochondrial cytochrome c oxidase subunit I (Cox1) PhyML tree of polynoids based on a 658‐bp alignment of 557 sequences. Listed next to the tree are the three main geographic areas, under which are the numbers of sequences for each lineage. All lineages are exclusively found at a single geographic area, except for *H. fuligineum* and the taxon determined as *Eunoe* sp. (arrows). Posterior probability values for the nodes are provided for all terminal clusters. Species names have been emphasized for only those taxa that had enough sequences (*n* > 30) for haplotype network investigations

We have assembled the largest known phylogenetic dataset of Antarctic polynoids collected from regions around the Antarctic continent, sub‐Antarctic, and Tierra del Fuego archipelagos. The amplification success was high (ca. 94%) for samples collected less than a year before DNA extraction (most Adélie Land and Ross Sea samples) and decreased as storage time increased (e.g., amplification success was 85% after 6 years for Kerguelen samples). With these samples and additional data from Amundsen Sea, New Zealand, Argentinean seamounts, and the Indian Ocean (Figure 2), we produced sequence alignments for two mitochondrial genes, Cox1 (a total of 691 sequences, including previously published ones) and 16S (a total of 219 sequences). The Cox1 dataset provided the most valuable insights into the evolutionary history of polynoids (Figure 3), while 16S often did not differentiate closely related taxa (Figure A1). Specifically, initial sequence data analyses revealed that Cox1 was able to discriminate species that 16S could not; *Harmothoe crosetensis* and *H. fuligineum* have identical 16S sequences but can easily be distinguished based on Cox1 sequences (Figure 3; Figure A1). Similar observations can be made for *Gorekia crassiciris* and *Gorekia* sp., and *Polyeunoa laevis* clusters 1 and 2 (Figure A1). Internal transcribed spacer 2 (ITS2) was also attempted in polynoids from other environments; however, the presence of microsatellites with intragenomic repeat number diversity prevented direct sequencing of this variable nuclear marker (S. Hourdez, observation). Despite this, Cox1 and 16S sequences produced in this study are publicly available at GenBank under accessions MT138932–MT139461 and MT139654 ‐ MT139872 for each dataset, while Cox1 accession numbers and locations are provided in Table A3. As such, the following results focus only on the Cox1 dataset as this marker provides the best resolution of those taxa investigated thus far.

### 3.1 Geographic distribution

The Cox1 dataset produced in this study contained a total of 691 sequences from the Antarctic (including Amundsen Sea, Adélie Land, Peninsula, Ross Sea regions, *n* = 599), the Kerguelen sub‐Antarctic Islands (*n* = 72), and Tierra del Fuego archipelago at the tip of South America (*n* = 16), as well as sequences from the Argentinean seamount (*n* = 5) and Indian Ocean (*n* = 4) (see Table A2 for the numbers of sequences specific to each geographic location and study). The sequences generated matched only to complementary queries in GenBank having Antarctic and South American origins (i.e., there were no sequence hits to non‐expected locations).

The dataset described herein distinguishes 36 lineages across the three broad geographic ranges (Figure 3). Out of this dataset, the five most abundant species (*B. cristata*, *H. crosetensis*, *H. fuligineum*, *H. magellanica*, and *P. laevis*) represent 91% of the sequences. Although some of the remaining sequences matched hits from published studies on Antarctic annelids, most corresponded to rare species (1–2 sequences) in our dataset that had no match in GenBank.

The inclusion of Tierra del Fuego specimens in the dataset allowed us to identify a single individual originating from Kerguelen whose sequence is identical to that of the most common taxon collected from Tierra del Fuego, determined as *Eunoe* sp. (Figure 3). Another single specimen that originated from the Kerguelen dataset was identified as *H. fuligineum*. *H. fuligineum* is otherwise only found at Antarctic continental regions where it is the most abundant species (Figure 3). All other species are exclusively found at a single geographic area: either the Antarctic continent, Kerguelen, or Tierra del Fuego.

Species accumulation curves indicate that the Antarctic continental dataset reaches a plateau at approximatively 28 species (Figure A2a), including 23 species from our sequence dataset, and the remaining five represented species present in GenBank. In contrast, the Kerguelen dataset does not reach a plateau, identifying only seven Polynoidae species revealed so far, and the Kerguelen accumulation curve remains well below that of the continent (Figure A2a). Additionally, relative abundance distributions along the Antarctic continental region reveal the presence of three abundant species, *H. crosetensis*, *B. cristata*, and *H. fuligineum*, followed by several rarer species (Figure A2b). The Kerguelen dataset exhibits a similar pattern of relative abundance, with few abundant species followed by other, less common species (Figure A2b). Given the small number of specimens obtained from Tierra del Fuego, we were unable to assess the relative abundance or species diversity of that location at this time.

### 3.2 Haplotype networks

**FIGURE 4:**
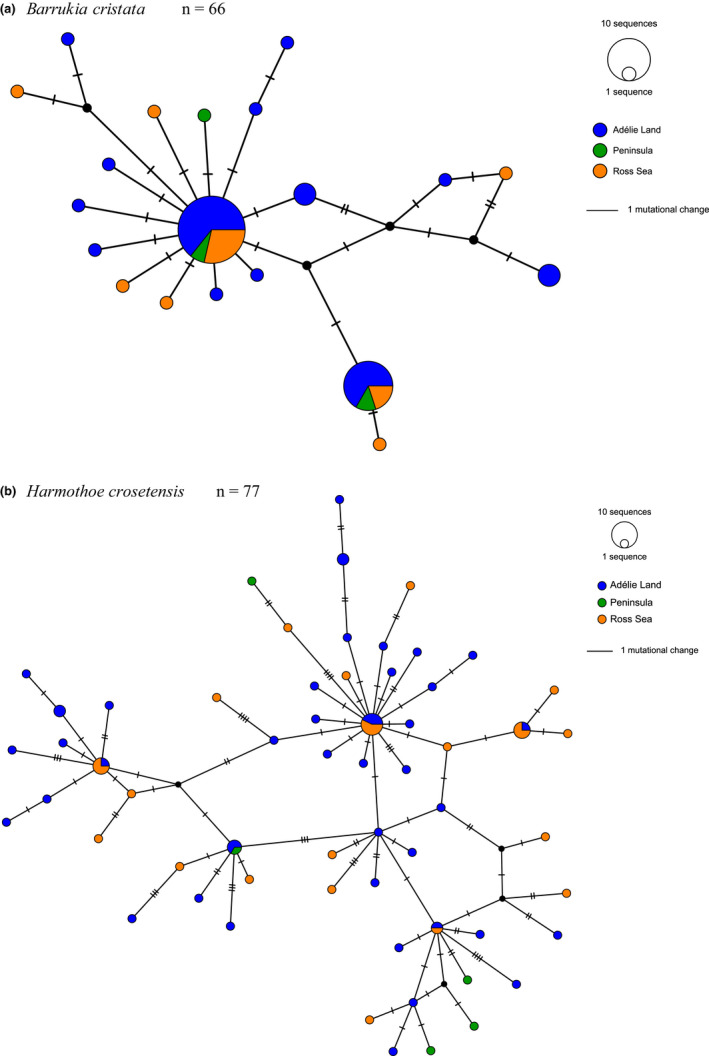
Haplotype networks based on Cox1 sequences for (a) *Barrukia cristata* (*n* = 66) and (b) *Harmothoe crosetensis* (*n* = 77)

*Barrukia cristata* and *H. crosetensis* individuals were collected only from the Antarctic continent at Adélie Land, Peninsula and Ross Sea regions (Table 1); alignments for both species were 658‐bp in length and the number of phylogenetically informative sites for *B. cristata* and *H. crosetensis* numbered 21 and 60, respectively. The *B. cristata* network was characterized by two common haplotypes, flanked by several low‐abundance haplotypes, sometimes unique to specific Antarctic continental regions (Figure 4a). The most common haplotypes were shared across the three regions, suggesting a homogeneous geographical distribution, which was supported by a nonsignificant Fst value (0.01789) and pairwise Fst values (Table 1, Hudson et al., 1992 Fst values). *H. crosetensis* displays a greater diversity of haplotypes, with five, more common haplotypes connected by complex relationships (Figure 4b). There was no overall geographic differentiation seen as the Fst value of 0.02272 was not significant (Table 1).

**TABLE 1:** Locations, number of sequences, Fst and pairwise Fst values for *Barrukia cristata*, *Harmothoe crosetensis*, *Harmothoe fuligineum* and *Polyeunoa laevis* clusters 1 and 3, as cluster 2 had too few sequences for performing these analyses. *Harmothoe magellanica* is not shown given that it represents a local Kerguelen species. Alignment lengths were all 658‐bp. The Hudson et al. (1992) Fst values for each species are located under the species' name. The number of sequences for each species by region is identified within a separate column, while pairwise Fst values are reported in adjacent columns. (*) indicates significance at *p* < .05

	Number of sequences	Antarctic peninsula	Ross Sea	Adélie land
*Barrukia cristata* Fst = 0.01789	66			
Antarctic Peninsula	5	—		
Ross Sea	19	−0.00606	—	
Adélie Land	42	0.07394	−0.01300	—
*Harmothoe crosetensis* Fst = 0.02272	77			
Antarctic Peninsula	5	—		
Ross Sea	27	0.03483	—	
Adélie Land	45	0.02882	0.00161	—
*Harmothoe fuligineum* Fst = 0.00470*	163			
Antarctic Peninsula	19	—		
Ross Sea	38	0.00146	—	
Adélie Land	106	0.00415	0.00999	—
*Polyeunoa laevis cluster 1* Fst = −0.01020	78			
Antarctic Peninsula	5	—		
Ross Sea	59	0.02631	—	
Adélie Land	14	0.00000	0.00432	—
*Polyeunoa laevis cluster 3* Fst = 0.01813	109			
Antarctic Peninsula	38	—		
Ross Sea	37	0.02001	—	
Amundsen	34	0.00759	0.04276	—

**FIGURE 5:**
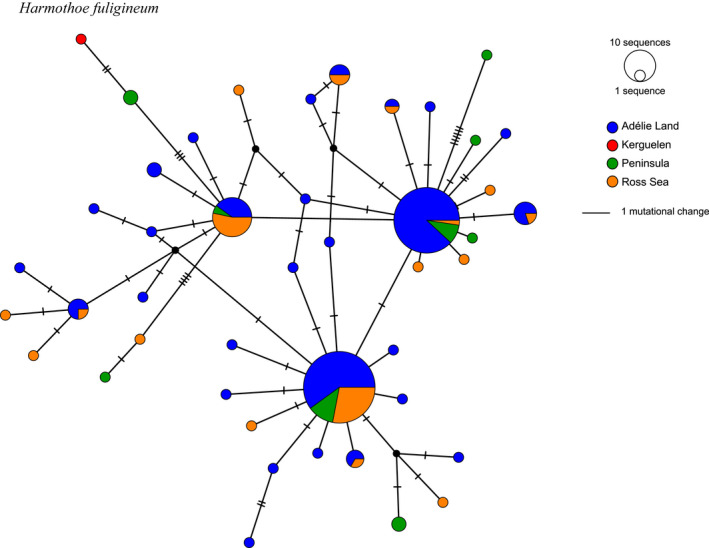
Haplotype network based on Cox1 sequences for *Harmothoe fuligineum* (*n* = 164)

The *H. fuligineum* network was comprised of individuals from each Antarctic region, as well as a single individual from Kerguelen. The length of the alignment was 658‐bp, of which 49 sites were phylogenetically informative. The *H. fuligineum* network reveals three common haplotypes, all found at each sampled locality (Figure 5). Two common haplotypes, nevertheless, are dominated by sequences from Adélie Land, while the remaining common haplotype is dominated by sequences from the Ross Sea. One common shared haplotype that is much less frequent in the Ross Sea compared with Adélie Land (Figure 5, χ^2^ *p* < .01). This geographic heterogeneity is supported by a Slatkin and Madison (1992) Fst value of 0.00470, significant at *p* < .05. Pairwise *F*st values ranged from 0.00161 between Ross Sea and Peninsula, and 0.00999 between Adélie Land and the Ross Sea (Table 1). Although the sequence from the lone individual from Kerguelen is most closely related to individuals from the Peninsula, it differs by two mutational changes (Figure 5). Lastly, the large number of collected *H. fuligineum* individuals allowed us to perform χ^2^ testing for possible intraspecies differences across a bathymetric depth range of 0–400 m; however, no significant depth‐related differences were detected within the dataset (not shown), but this could be masked by the regionalization we described above.

The species *H. magellanica* was only collected from Kerguelen, yielding an alignment length of 658‐bp, of which 23 sites were phylogenetically informative. Although *H. magellanica* represents a local Kerguelen species, a network was produced to illustrate a single dominant haplotype among several, less common variants (Figure A3).

**FIGURE 6:**
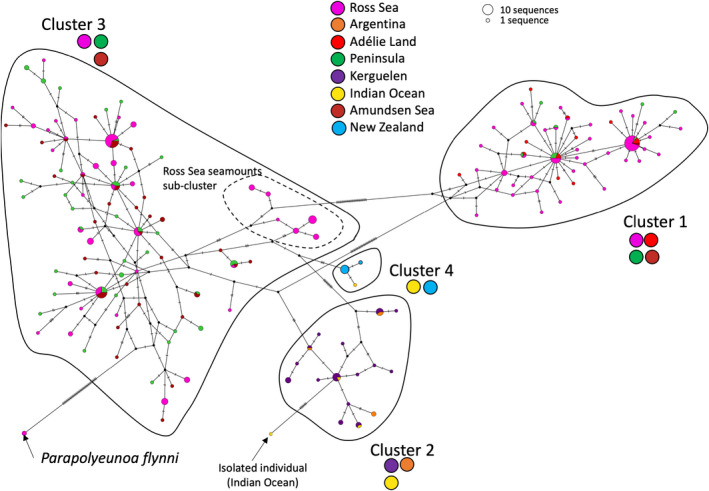
Haplotype network based on Cox1 sequences for *Polyeunoa laevis* (*n* = 274). The closely related species *Parapolyeunoa flynni* is included for reference. For each cluster, the colored stickers below the name summarize the regions of the sequences present in the cluster

*Polyeunoa laevis* was obtained from various localities, including Adélie Land, Peninsula and Ross Sea continental regions, as well as from Kerguelen. Sequences from the Amundsen Sea, Argentinean seamounts, Indian Ocean, and New Zealand (Brasier et al., 2017; Bogantes et al., 2020; Serpetti et al., 2017; Schiaparelli unpublished, see Table A2) were also added to this analysis. The length of the alignment was 658‐bp, of which 103 sites were phylogenetically informative. The *P. laevis* network reveals a clear subdivision of the morphological species into three major clusters, separated by an accumulation of mutations and the overall co‐occurrence of 4 clusters (Figure 6). Cluster 1 is dominated by haplotypes originating from Antarctic continental regions (Adélie Land, Amundsen Sea, Peninsula, Ross Sea), while Cluster 2 consists of haplotypes originating from sub‐Antarctic regions (Kerguelen, Argentina) and the Indian Ocean. Cluster 3 is comprised of haplotypes from the Antarctic (Peninsula, Ross, and Amundsen Seas). Cluster 4 includes only sequences that do not correspond to specimens from Antarctica or sub‐Antarctic regions (Indian Ocean and New Zealand).

Cluster 1 identifies the most common haplotype as shared across all Antarctic localities, while cluster 2 identifies two haplotypes that are shared between the Kerguelen and the Indian Ocean, signifying an ongoing exchange between these two regions that are in proximity to each other (Figure 6). Furthermore, this cluster also identifies shared haplotypes between Kerguelen and Argentina. Cluster 3 characterizes multiple common haplotypes as being shared across Peninsula and the Ross and Amundsen Seas, as well as a subcluster of haplotypes emerging from seamounts located in the Ross Sea. Given the low number of sequences retrieved for clusters 2 and 4, Fst values were generated and tested only for clusters 1 and 3 (Table 1). For these clusters, there was no overall geographic differentiation, showing nonsignificant Fst values (Table 1).

### 3.3 Genetic diversity and past demographic changes

Based on regionalization results and detection of cryptic species, the following indices calculations were made for the entire datasets of *B. cristata*, *H. crosetensis*, and *H. magellanica*, for the three localities of *H. fuligineum*, as well as the three main clusters (potential cryptic species) we identified for *P. laevis*.

*Barrukia cristata*, *H. crosetensis*, *H. fuligineum*, and *H. magellanica* display a high haplotype diversity (Hd 0.765–0.987) and a low‐to‐moderate nucleotide diversity (π 0.0027–0.0091) (Table 2). In the range of values, *B. cristata* and *H. fulgineum* (Adélie Land) have both the lowest haplotype diversity and the lowest nucleotide diversity, while *H. crosetensis* had the highest values for both parameters. Primarily, Antarctic continental species *B. cristata*, *H. crosetensis*, and *H. fuligineum* displayed significant deviations from the neutral accumulation of mutations in their polymorphisms, with negative Tajima's D and Fu & Li values indicating an excess of rare haplotypes. *H. fulgineum* (Peninsula) and *P. laevis* cluster 2 both have a relatively small number of sequences (*n* = 19 and *n* = 25, respectively) and nonsignificant Tajima's D and Fu & Li values. The presence of at least three cryptic species within *P. laevis* called for the individual analyses of each cluster, each of which displayed high haplotype and nucleotide diversities (0.0059–0.01022, Table 2). Significant Tajima's D and Fu & Li values, however, were only obtained for the continent‐based clusters 1 and 3 (Table 2).

**TABLE 2:** Nucleotide diversity of five species investigated using haplotype networks. *P. laevis* is subdivided into its three major clusters illustrated in Figure 6; cluster 1: Adèlie land/Peninsula/Ross Sea/Amundsen Sea, cluster 2: Kerguelen/Indian Ocean/Argentina, cluster 3: Peninsula/Ross Sea/ Amundsen Sea. Cluster 4 is not included given its low number of sequences

Species	Ns	Nh	Hd	π	Tajima's D	Fu & li′s D	Expansion test Δχ^2^ df	Tau	Calculated age (kya)
*Barrukia cristata*	66	20	0.765	0.00302	−1.93459*	−3.26372*	969.9 19 ***	1.38	59.0
*Harmothoe crosetensis*	77	60	0.987	0.00910	−1.94267*	−3.69112**	4394 22 ***	4.48	191.4
*Harmothoe fuligineum* (Adélie Land)	106	28	0.797	0.00270	−2.10538*	−3.57024**	3396.2 19***	1.52	65.0
*Harmothoe fuligineum* (Peninsula)	19	9	0.866	0.00560	−1.62137^NS^	−1.71020^NS^	25.7 25 NS	(1.26)^a^	(53.8)^a^
*Harmothoe fuligineum* (Ross Sea)	38	17	0.829	0.00334	−1.94000*	−3.64615**	671.7 19 ***	1.83	78.2
*Harmothoe magellanica*	33	17	0.877	0.00653	−0.84029^NS^	−1.76988 ^NS^	5936.1 19 ***	1.10	47.0
*Polyeunoa laevis* cluster 1	79	45	0.918	0.00595	−2.22592***	−4.09878 **	3972 19 ***	2.92	124.8
*Polyeunoa laevis* cluster 2	25	15	0.940	0.00752	−0.63838 ^NS^	−0.10357 ^NS^	364.98 20 ***	2.54	108.5
*Polyeunoa laevis* cluster 3	114	77	0.981	0.01022	−2.00242*	−4.7969 **	9257 22 ***	5.01	214.1

Abbreviations: df, degrees of freedom; Hd: Haplotype diversity; Nh: Number of haplotypes; Ns: Number of sequences; Δχ^2^: difference of distribution of observed pairwise distances compared with a constant site population and an expanding population; π: Nucleotide diversity.

^a^ This identifies the value as only indicative, as expansion is not demonstrated.

Statistical significance level: * < 0.05, ** <0.02, *** <0.001, NS = not significant.

The test of expansion of populations was highly significant for all species and populations, except for *H. fulgineum* (Peninsula) (Table 2). This allowed us to estimate Tau, provided by the curve fitting on pairwise distance distributions in DnaSP, for all and calculate the corresponding estimated ages of the last bottleneck ranging from 47 to 214.1 kya. The range was relatively small for the populations of *H. fuligineum* (65.0–78.2 kya) but markedly larger for the three clusters of the cryptic species *P. laevis* (108.5–214.1 kya).

## 4 DISCUSSION

### 4.1 Endemism and diversity of Antarctic polynoids

During our study, we found no molecular evidence of shared species between Antarctica and other regions of the world. Although we collected a Kerguelen specimen very closely related to *H. fuligineum* from the continent, its haplotype is unique. This is support for the notion of a long history of isolation of Antarctic marine fauna (Clarke et al., 2005), which is progressively recognized in studies based on molecular data where new subfamilies (Barco et al., 2012) or genera (Fassio et al., 2020) are established to accommodate Southern Ocean taxa previously grouped, based on morphology only, with non‐Antarctic counterparts. At the broadest geographic scale, none of our sequences matched entries from outside of the Antarctic or adjacent regions, despite the presence of approximatively 200 polynoid species having Cox1 sequences available in GenBank, out of about 900 described species worldwide. Some sequences for the very common species *Harmothoe fuligineum* did not match conspecifics previously present in GenBank, possibly due to the presence of cryptic species or misidentifications based on morphological determinations.

There is furthermore a clear separation between the Antarctic continent, Kerguelen, and Tierra de Fuego, with two noticeable exceptions: (1) one specimen from Kerguelen shared a haplotype sequence with the most common *Eunoe* sp. in the Tierra de Fuego dataset and (2) another specimen from Kerguelen produced a sequence that was nearly identical to sequences from Antarctic continental lineage *H. fuligineum* (Figure 3). The first finding provides evidence for a probable ongoing or recent connectivity between South America and Kerguelen, possibly driven by the clockwise flow of the Antarctic Circumpolar Current (ACC) (González‐Wevar et al., 2021; Pfuhl & McCave, 2005). The presence of *H. fuligineum* at Kerguelen could be indicative of present‐day gene flow but also the signature of past connectivity between this archipelago and the Antarctic continent during the last maximal glacial extension occurring about 18,000 years ago (Rogers, 2007). During ice extension, benthic populations frequently find refuge in areas free of ice, either in shallow habitat of sub‐Antarctic islands or in deep water. As a result, populations can genetically diverge into separate species; when these groups are brought back into contact during ice retreat (“secondary contact”), they may remain genetically isolated or undergo hybridization. Extension and retreat of ice has been invoked to help explain the present‐day distributions of species (Hemery et al., 2012). Whether present‐day exchange still occurs between the Antarctic and the sub‐Antarctic islands will require more precise population genetic studies of *H. fuligineum*. This calls for additional efforts to sample this morphospecies in Kerguelen to document the Cox1 haplotype diversity there. With a larger sample dataset, we may be able to eventually bridge the mutational gap with the other haplotypes or confirm the barcode gap. A focused study with finer populational makers such as microsatellites or RADseq would be very useful in shedding light on past populational changes and the effect of ice extension. Further sampling from other sub‐Antarctic islands would also be very useful for our understanding of the evolutionary history of this species.

Based on rarefaction curves, most of the contemporary polynoid species diversity has been depicted around the Antarctic, including 28 distinct Cox1 lineages (Figure A2a). At Kerguelen, seven distinct lineages were uncovered, and the rarefaction curve did not reach a plateau (Figure A2a). The 16S dataset is more limited and comprises 16 lineages, some of which correspond to what was recognized as two lineages for Cox1 (Figure A1). Therefore, we have uncovered a portion of the total existing diversity, compared to approximately 50 species of polynoids currently described from the Southern Ocean (De Broyer et al., 2022). It is important to note that most of our sampling efforts were performed in <200 m depth, across differing research expeditions with various objectives and technical limitations. In addition, every individual collected did not yield a Cox1 amplification, all of which may account for missing diversity. Moreover, it must also be considered that *Polyeunoa laevis* was specifically investigated to unravel its possible cryptic diversity, and hence was overrepresented in the available samples.

Among the lineages, some subfamilies that are common in other environments are conspicuously missing in our samples and could indicate their complete absence in Antarctica. These absences were confirmed by both molecular and morphological identifications. All subfamilies identified based on the morphology of the specimens are represented in the molecular dataset. The Lepidonotinae are common in shallow water environments (one specimen, collected in Tierra de Fuego) but were not found in our Antarctic samples. Similarly, the Iphioninae (now considered a family, Iphionidae) are common in tropical waters, on seamounts, and near deep‐sea hydrothermal vents (McCowin & Rouse, 2018; Wehe, 2017), but completely missing in our collections.

### 4.2 Species regionalization around the Antarctic continent

Of all the species with multiple populations sampled around the Antarctic continent, only *H. fuligineum* revealed regionalization to some extent; the Ross Sea population exhibited differences in some haplotype frequencies compared to the Adélie Land and Peninsula populations (Figure 5). Other species in our study are relatively rare and the absence of a lineage from a region could reflect a sampling bias rather than its actual absence in the environment.

Except for *H. fuligineum*, polynoid lineages collected from regions thousands of kilometers apart (Table A3) displayed almost no genetic differentiation around Antarctica, which has been previously observed in Antarctic species with a high dispersal capacity (Allcock & Strugnell, 2012). High dispersal is a common trait of broadcast spawners, which includes many polynoids (Daly, 1972; Giangrande & Petraroli, 1994; Plyuscheva et al., 2004). In addition, previous surveys have provided support for the presence of polynoid broadcast spawners; these studies performed plankton sampling during the austral summer which captured larvae with sequences matching *H. crosetensis* (GenBank accession GU227143) and *H. fuligineum* (GenBank accession GU227137) (Heimeier et al., 2010). Additionally, larvae (including nectochaetes) morphologically identified as polynoids were collected near the Dumont d'Urville station (Adélie Land) and in the Ross Sea (Bhaud et al., 1999). Combined, high dispersal exhibited by broadcast spawning strategy is closely linked to species having higher gene flow and less geographic fragmentation (Slatkin, 1987) presently seen with *B. cristata* and *H. fuligineum*.

### 4.3 Genetic diversity, local demography, and evolutionary history of species

Expansion analyses performed showed that all species and populations considered display characteristics of expanding populations, except *H. fuligineum* from the Antarctic Peninsula, for which the small number of sequences could have led to this lack of significance (Table 2). These findings are corroborated for most by significant negative Tajima's D and Fu & Li values, which indicate an excess of rare haplotypes that typically occurs during the expanding period after a bottleneck event within these species, or either background selection or selective sweeps acting more specifically on the mitochondrial genome (Depaulis et al., 2003; Galtier et al., 2009). The bottleneck event was possibly driven by habitat reduction resulting from ice expansion during ice ages that drastically reduced populations in some specific refuges and their genetic variability (Rogers, 2007). The species *H. magellanica* also displays evidence of expansion yet is only found on Kerguelen (Figure A3). This could indicate that the ice expansion could have been sufficiently far‐reaching to affect sub‐Antarctic species and result in drastic temperature changes.

All calculated bottleneck ages range from 47 to 214.1 kya (Table 2), well in the mid Pleistocene during which extensive glaciations occurred on a 100,000‐year cycle (Hasenfratz et al., 2019). Of the three Antarctic continental species, nucleotide diversity and calculated age as the bottleneck was highest for *H. crosetensis* (191.4 kya) (Table 2). In our dataset, expansion ages this old are only found in the potential species complex *P. laevis*, which are known from depths below 150 m. It is possible that *H. fuligineum* and *H. magellanica* populations were more recently affected compared to *H. crosetensis* populations, which possibly maintained connectivity by migrating into deeper waters and later colonizing shallower environments.

### 4.4 Polyeunoa laevis: Potential movement across depths and along the APF

*P. laevis* is reported from the Southern Ocean at depths greater than 150 m and frequently found on seamounts worldwide; however, larval stages corresponding to the two Antarctic lineages of *P. laevis* (Cox1 barcoding) have been collected in shallow plankton trawls in the Ross Sea (Heimeier et al., 2010). Recent studies have pointed to the existence of important morphological variations and possible cryptic lineages existing within this taxon (Alvaro et al., 2014; Barnich et al., 2012; Serpetti et al., 2017). In this study, we uncovered evidence for at least three cryptic species which also represent distinct geographic groups. The divergence level between these groups (11–29 fixed mutations, Figure 6) corresponds to 1–2.5 million years divergence, about 10 times the age of the expansion for each cluster (Table 2). Altogether, these observations suggest that deep occurring groups such as *P. laevis* can also be impacted by glaciations, and thus geographic diversification.

Cluster 2 identifies the presence of a shared haplotype between Kerguelen and Indian Ocean, demonstrating that the Kerguelen Archipelago represents a possible stepping‐stone from the Indian Ocean toward the Antarctic continent (Figure 6). Furthermore, cluster 2 identifies shared haplotypes between the Kerguelen archipelago and Argentinean seamounts, suggesting a possible transport of this lineage from east toward Kerguelen, following the APF. Tracing the complex history of *P. laevis* outside the Antarctic continent will require additional sequences from seamounts south and north of the APF, particularly near South America and other sub‐Antarctic archipelagos such as Prince Edward Island where the *P. laevis* holotype has been described (Bogantes et al., 2020). Southern Indian Ocean sequences provided by (Serpetti et al., 2017) have helped to reveal a group of haplotypes shared with samples from Kerguelen and uncovered two other genetically distinct individuals separated by 9 mutational steps (cluster 2, Figure 6). Additional cryptic species could therefore be discovered from an even broader sample inventory. Furthermore, cluster 4 (New Zealand and Indian Ocean haplotypes) supports that even at depths greater than 150 m, there is a clear separation between Antarctic specimens and specimens from other locations. Lastly, another possible explanation for the apparent divergence and co‐speciation of *P. laevis* is its symbiotic relationship with various species of deep‐sea gorgonians (Anthozoa: Gorgoniidae) (Bogantes et al., 2020; Serpetti et al., 2017) as well as other soft corals and stylasterids (Schiaparelli, observation) which may have represented the basal driving force.

### 4.5 The Kerguelen archipelago as a crossroads

The presence of (1) a unique species at Kerguelen (*H. magellanica*), (2) one shared between Kerguelen and Tierra del Fuego (*Eunoe* sp.), and (3) one shared between Kerguelen and the Antarctic continent (*H. fuligineum*) (Figure 3) suggest that the Kerguelen Archipelago could act, or have acted in the past, as sink for larvae exported from these two other locations. Although within the intraspecific nucleotide differences of the Antarctic continental populations of *H. fuligineum*, the haplotype sequence of the Kerguelen specimen differs by at least two specific mutations from the other haplotypes (Figure 5), identifying that this individual is genetically distinct from the wider group. However, despite having larger numbers, the haplotypic diversity of *H. fuligineum* was not fully uncovered and we cannot rule out the possibility that the Kerguelen haplotype is not present in the Antarctic continental populations. Modeling particle transport from the continent demonstrated that larvae could indeed reach sub‐Antarctic islands such as those found in the Kerguelen Archipelago (Brasier et al., 2017). The possibility of transport in the reverse direction, however, was not investigated. As a result, it remains unclear whether species from Kerguelen could eventually colonize the Antarctic continent if regional warming trends continue (Frenot et al., 2006).

### 4.6 Implications of warming climate on Antarctic benthic communities

Recent warming trends in the oceanic province surrounding Antarctica will continue to impact biological communities at all life stages by continued physical modification of benthic habitats and abiotic parameters (temperature, pH, salinity) (Barnes & Conlan, 2007; Ingels et al., 2012). Understanding the history of benthic species and modern gene flow patterns is therefore necessary for elucidating how continued environmental changes will influence overall ecosystem functioning in Antarctica.

When compared with plankton and nekton, the marine benthos is especially vulnerable to habitat alterations, as warming temperatures increase iceberg grounding and scouring that mechanically disturbs these habitats. Benthic organisms encounter difficulties escaping these perturbations and to recolonize these damaged areas (Gutt, 2001; Gutt et al., 1996). Physical disturbances linked to warming can be particularly devastating for organisms living at shallower depths (< 400 m, Lee et al., 2001, Gerdes et al., 2003; Gutt & Starmans, 2001). Conversely, some Antarctic benthic invertebrates show adaptive characteristics and resilience to frequent and seasonal ice disruptions, including higher environmental tolerances and wider geographic ranges (Barnes et al., 2010). Furthermore, each of the abundant species studied here have larvae with high dispersal capabilities, demonstrated by their broad geographic distribution; therefore, when one population is effaced, the same species is often present elsewhere.

Trends of warming are most stark on the west Antarctic Peninsula (Meredith & King, 2005) where the two most abundant polynoid species in the Antarctic, *H. fuligineum* and *H. crosetensis*, are present. The presence of *H. fuligineum* in the Kerguelen archipelago would suggest that this species may tolerate warmer temperatures on the peninsula. Assessing genetic polymorphism of polynoid species, and especially *H. fuligineum*, with additional genetic markers may improve our understanding of their adaptive potential to warming temperatures. This is especially important given the high temperature stability of the Antarctic waters over the last 10 million years that may have driven the loss of polymorphism by directional selection and frequent bottlenecks during glacial episodes (*see* Papot et al., 2016, *Euphausia* krill).

In summary, findings in the present study do not support a single origin for contemporary Antarctic polynoids, but some species investigated here provide information on ancestral scenarios of (re)colonization. It is apparent that species collected from the Antarctic continent are endemic. For most lineages, the absence of closely related species in the Kerguelen and Tierra de Fuego, as well as in Genbank, argues in favor of local origin of the polynoid species (i.e., no ongoing arrivals from South America). *Eunoe* sp., and *H. fuligineum*, however, support the possibility of Kerguelen (and other sub‐Antarctic islands) acting as a crossroads for some species of larvae. The genus *Polyeunoa*, conversely, found at depths greater than 150 m may have a deep origin. These “non endemic” groups, nevertheless, have a distribution that is either north or south of the Antarctic Polar Front, indicating that there is a barrier to dispersal even in the deep, and that this specific lineage could correspond to a scenario of colonization from the deep sea. Like many other benthic fauna, polynoid evolutionary history was also greatly affected by the succession of glaciation and thawing cycles that the Antarctic has experienced over its history.

## AUTHOR CONTRIBUTIONS

**Dominique A. Cowart:** Data curation (lead); formal analysis (lead); investigation (lead); resources (supporting); validation (equal); visualization (lead); writing – original draft (lead); writing – review and editing (equal). **Stefano Schiaparelli:** Formal analysis (equal); funding acquisition (lead); investigation (equal); project administration (lead); resources (lead); supervision (lead); validation (equal); visualization (equal); writing – review and editing (equal). **Maria Chiara Alvaro:** Formal analysis (equal); investigation (equal); validation (equal); writing – review and editing (equal). **Matteo Cecchetto:** Formal analysis (equal); investigation (equal); validation (equal); writing – review and editing (equal). **Anne‐Sophie Le Port:** Formal analysis (equal); investigation (equal); writing – review and editing (equal). **Didier Jollivet:** Conceptualization (lead); formal analysis (supporting); funding acquisition (lead); methodology (equal); project administration (lead); resources (lead); supervision (lead); writing – review and editing (equal). **Stéphane Hourdez:** Conceptualization (lead); formal analysis (lead); funding acquisition (lead); investigation (lead); methodology (equal); project administration (lead); resources (lead); supervision (lead); validation (equal); visualization (lead); writing – original draft (equal); writing – review and editing (equal).

## ACKNOWLGMENTS

We are especially grateful to Karin Gerard (University of Magallanes, Chile), Marie‐Laure Guillemin (Universidad Austral de Chile), Melyne Hautecoeur (Muséum National d'Histoire Naturelle), Marc Eléaume (Muséum National d'Histoire Naturelle), and Melody Clark (British Antarctic Survey) for their contributions to this project that include the collection and sharing of samples. We would like to acknowledge the crew of the Tangaroa expedition “IPY‐CAML TAN0802” for logistic support during the cruise and we are indebted to the New Zealand Ministry of Fisheries (MFish) and NIWA (Wellington) for the financial support of the cruise and related study activities. Logistics were provided by Institut Polaire Paul Emile Victor (IPEV) program POLARIS to SH. We thank the staff at bases/stations at Dumont d'Urville and Rothera, as well as Dr. Julian Gutt (Alfred Wegener Institute) for the invitation to the AWI PS81 ANT‐XXIX/3 and the donation of samples to the Italian National Antarctic Museum (MNA). We would also like to thank Pierre Chevaldonné, dive partner of SH during the POLARIS expeditions and avid naturalist; his involvement was essential for the collection of samples and documenting biodiversity in the area. Finally, we thank Olivier Soubigou for his assistance with aspects of the manuscript. This research was supported by an EC2CO grant to SH (ANTARES) and a UIUC STEM fellowship to DAC. Funding was also obtained through the Italian National Antarctic Research Program (PNRA, www.pnra.it) projects PNRA16_00120‐A1 (TNB‐CODE), PNRA18_00078 (RossMODE) and the Census of Antarctic Marine Life (CAML) to SS.

## CONFLICT OF INTEREST

The authors declare that the research was conducted in the absence of any personal or financial relationships that could be construed as a potential conflict of interest.

## SIGNIFICANCE STATEMENT

We used scale worms as a representative group for Antarctic marine benthic animals to better understand their evolutionary history, and possibly predict how they could respond to warming temperatures. Using a dataset of over 600 DNA sequences, we were able to comprehend the evolutionary history of this group during past warming and cooling geological periods. These events had profound effects on their genetic diversity that are still detectable in present populations. The worms are isolated from those in the north by the Antarctic Circumpolar Current and are unlikely to colonize waters around the Antarctic, raising concerns for the future of this ecosystem facing global warming.

## Supporting information


Figure S1

Figure S2

Figure S3

Appendix S1

## Data Availability

These sequence data have been submitted to GenBank under accession numbers MT138932 ‐ MT139461 (Cox1) and MT139654 ‐ MT139872 (16S) at www.ncbi.nlm.nih.gov/genbank. Dryad data can be found at https://doi.org/10.5061/dryad.p2ngf1vt0.
