# Supplementary figures and images for: Origin, diversity, and biogeography of Antarctic scale worms (Polychaeta: Polynoidae): a wide‐scale barcoding approach
Source: Ecol Evol. 2022 Jul 17;12(7):e9093. doi: 10.1002/ece3.9093 (PMC9288932; doi:10.1002/ece3.9093)

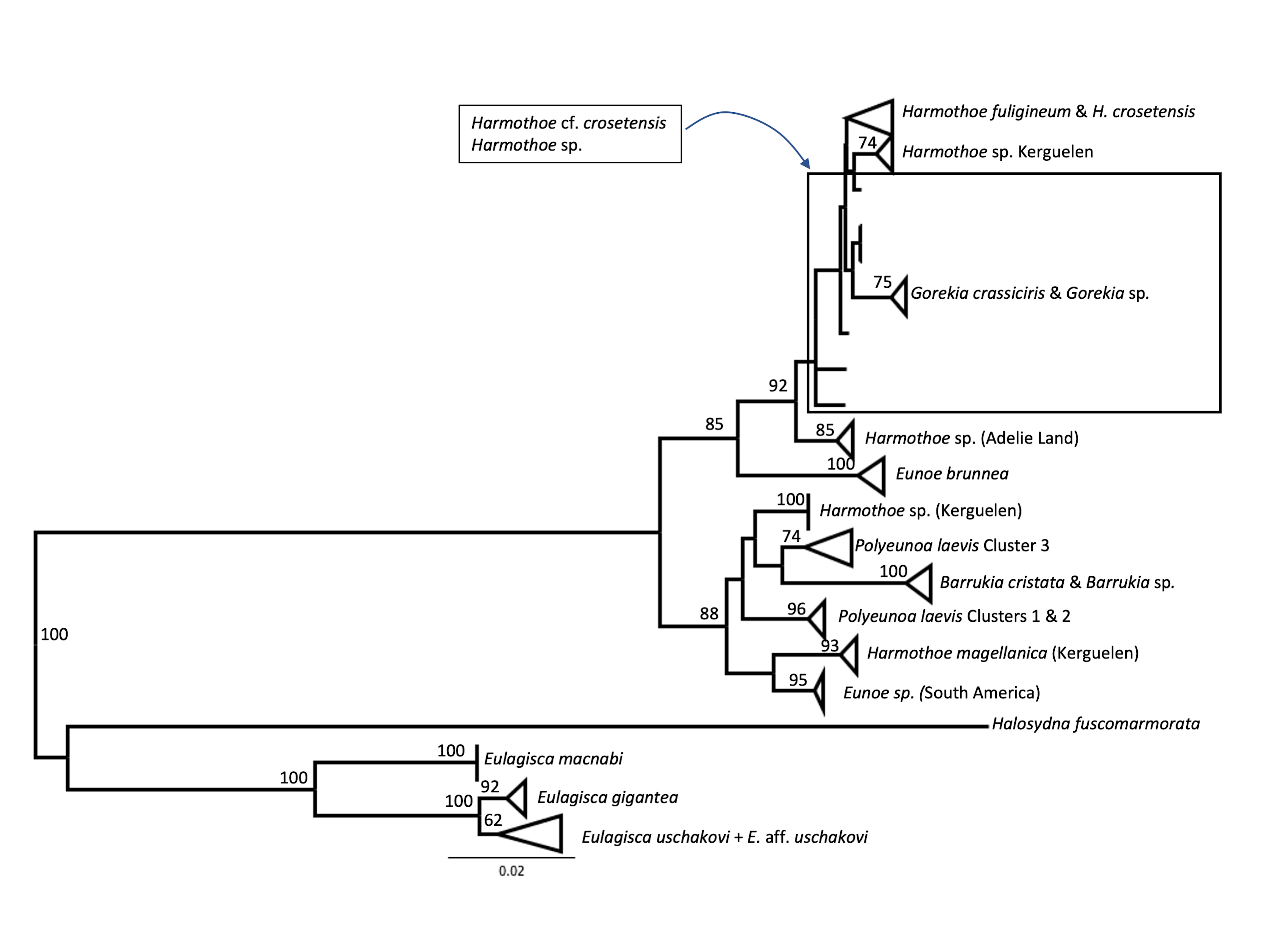

Supplement: Supplementary file 1 — Figure S1 [file ECE3-12-e9093-s004.jpg]

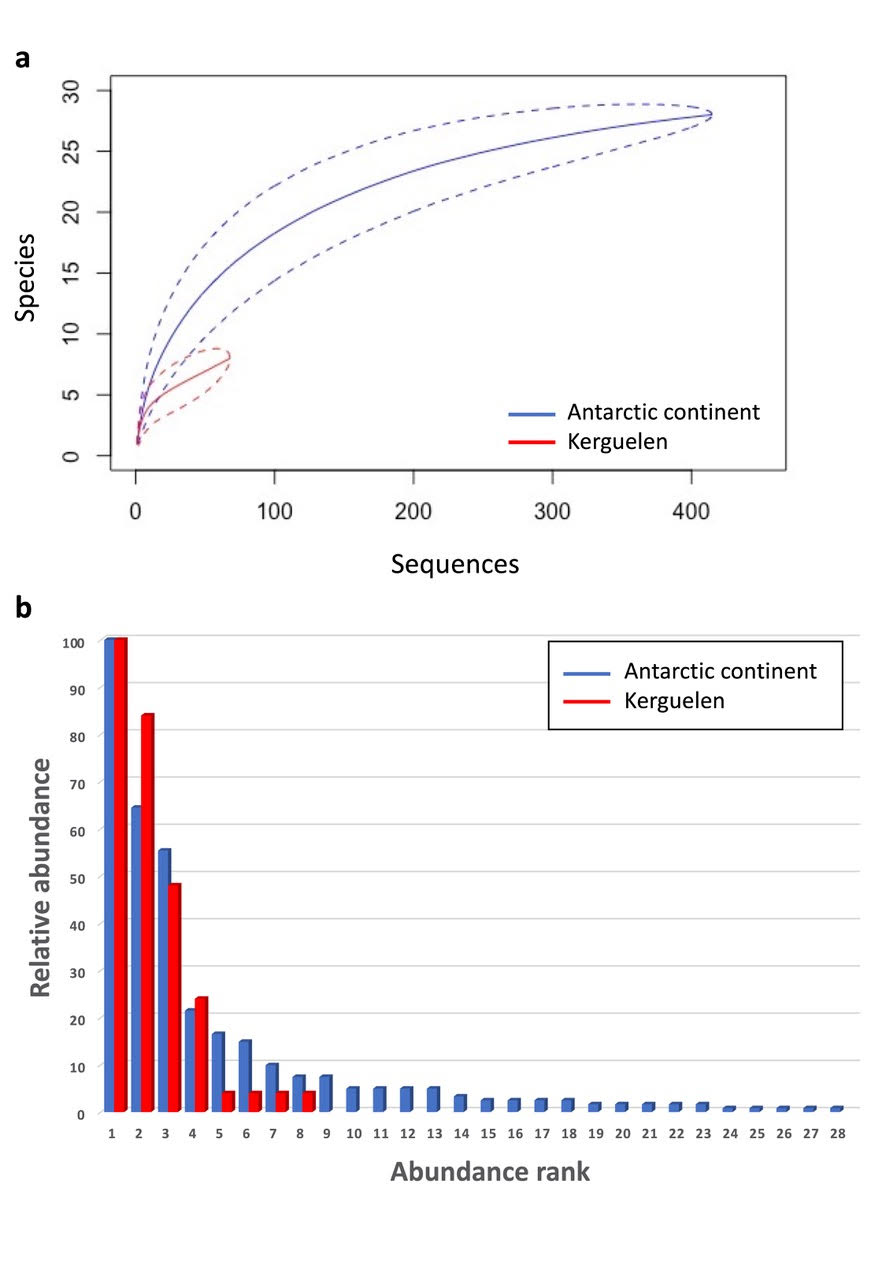

Supplement: Supplementary file 2 — Figure S2 [file ECE3-12-e9093-s003.jpg]

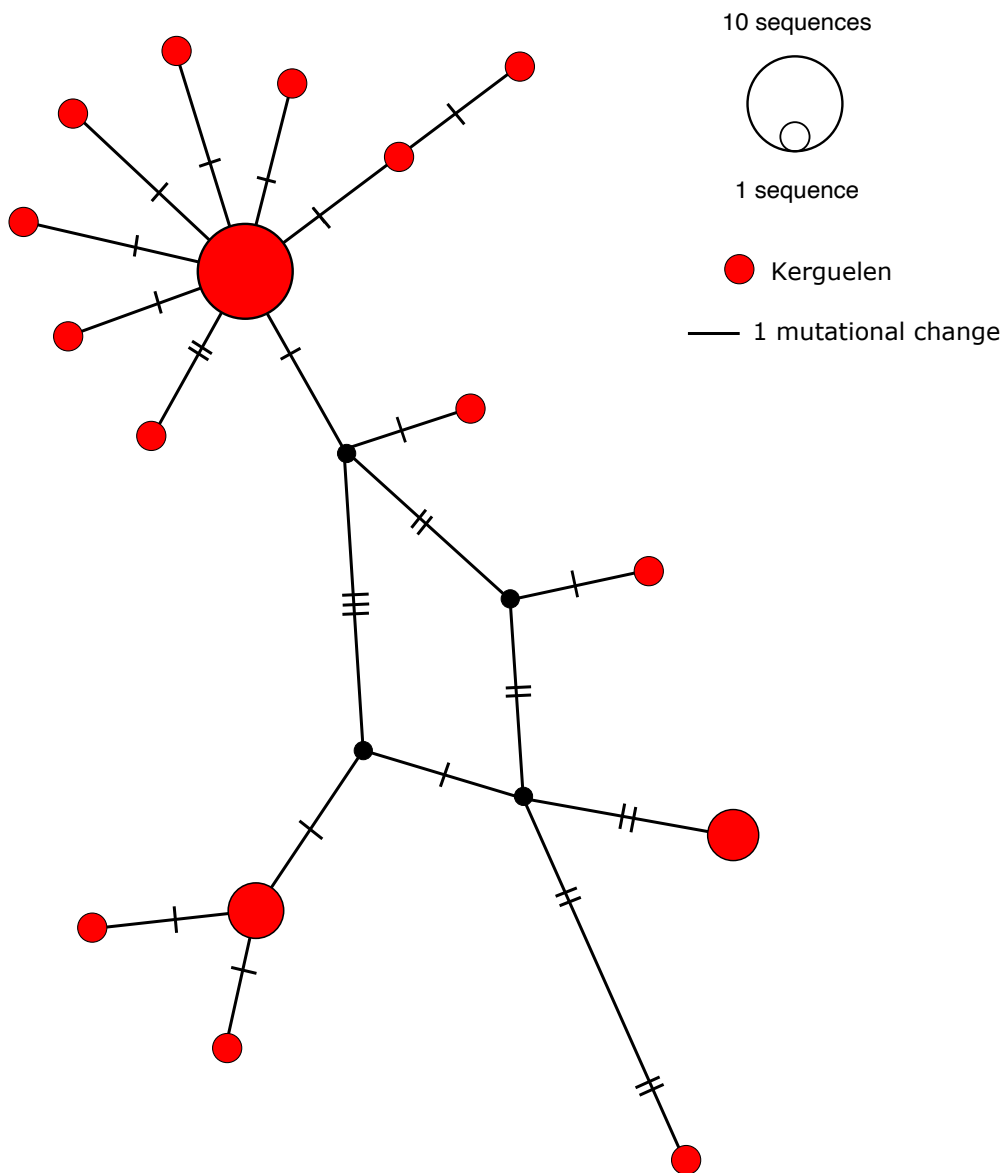

Supplement: Supplementary file 3 — Figure S3 [file ECE3-12-e9093-s002.pdf]
