# Supplementary material for: Origin, diversity, and biogeography of Antarctic scale worms (Polychaeta: Polynoidae): a wide‐scale barcoding approach
Source: Ecol Evol. 2022 Jul 17;12(7):e9093. doi: 10.1002/ece3.9093 (PMC9288932; doi:10.1002/ece3.9093)
Supplement: Supplementary file 4 — Appendix S1 [file ECE3-12-e9093-s001.docx]

**Appendix**

**Figures**

**Figure A1:** Mitochondrial 16S ribosomal RNA (16S) neighbor-joining phylogenetic tree based on a 512-bp alignment of 219 polynoid sequences, produced using K2-P distances. The numbers located over the branches are bootstrap values for 100 replicates; values below 50 have been omitted. The box indicates poorly-resolved nodes. Several lineages comprise distinct species (as identified based on Cytochrome c oxidase subunit I (Cox1) data, Figure 3) that cannot be resolved with this marker.


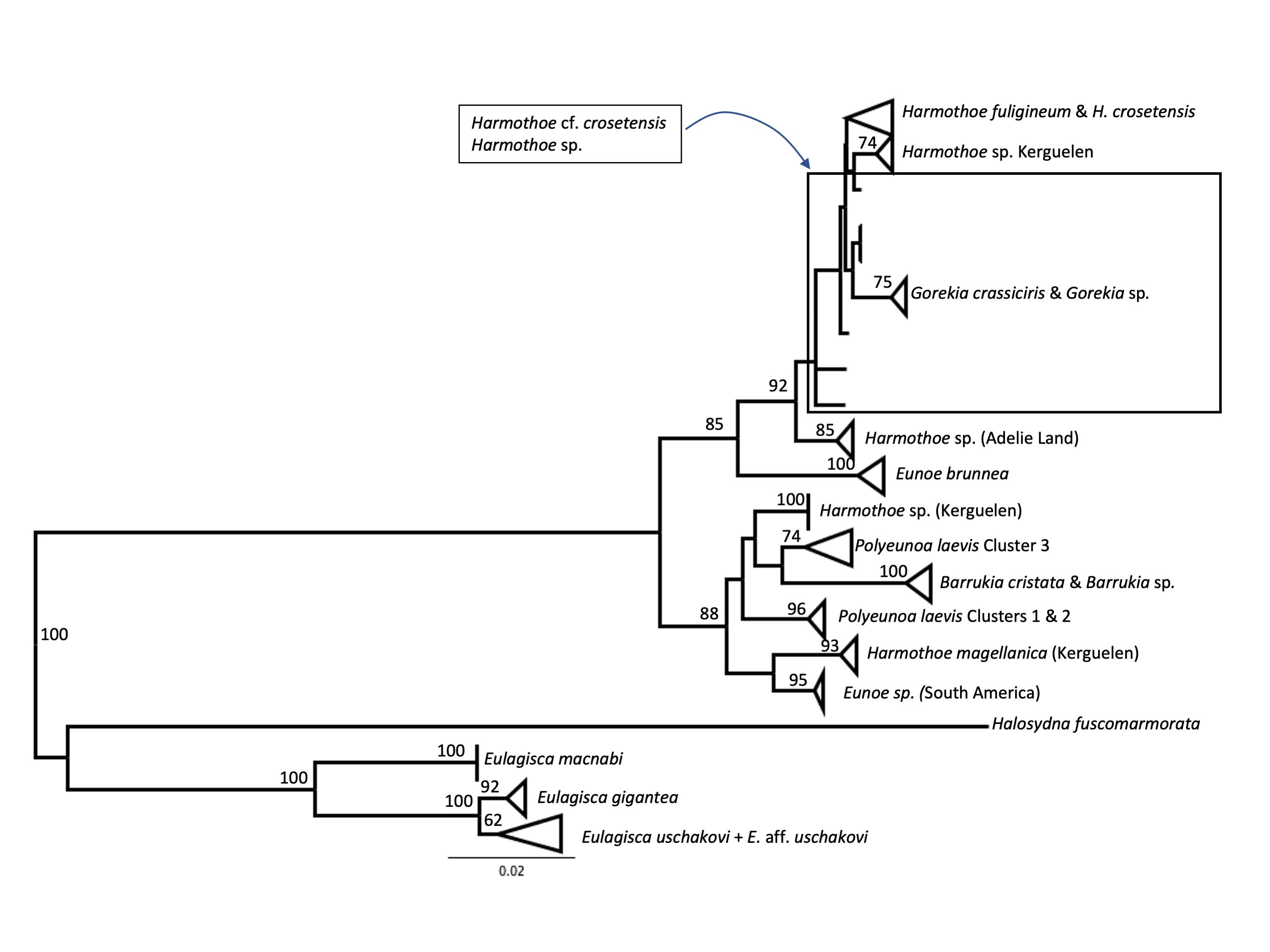


**Figure A2:** (a) Species accumulation curves as a function of sequencing effort. The dashed lines correspond to the 95% confidence intervals. (b) Relative abundances of species normalized using the most abundant species for each region.

**Figure A3:** Haplotype network based on Cox1 sequences for *Harmothoe magellanica* (n = 33).


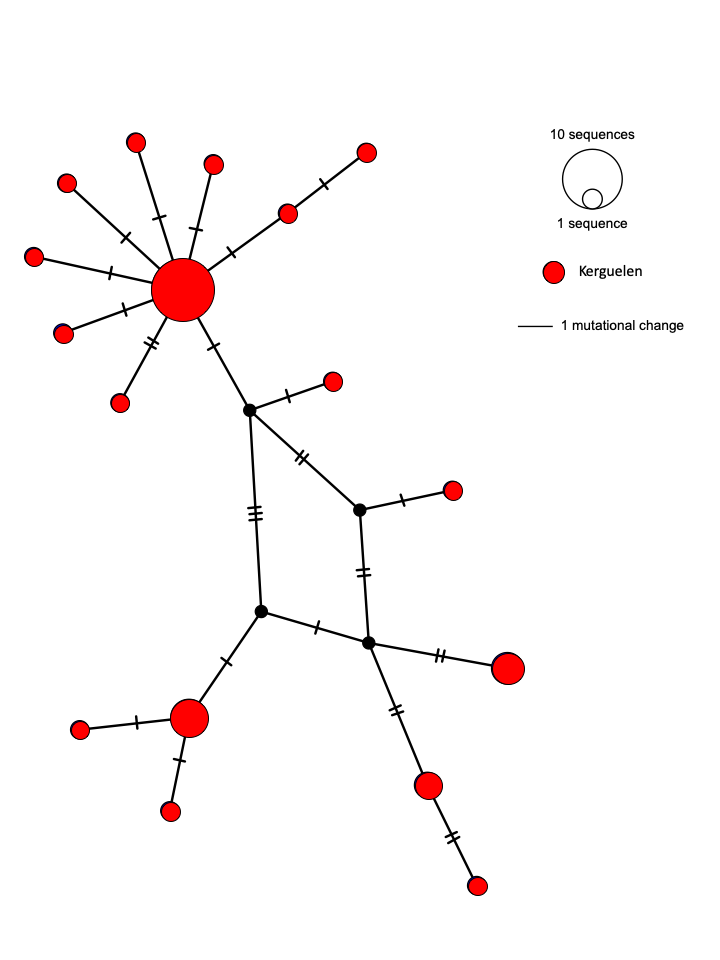


*Harmothoe magellanica* n = 33

**Tables**

**Table A1**: Mitochondrial Cytochrome c oxidase subunit I (Cox1) and 16S ribosomal RNA (16S) primers used in the present study.

| **Gene** | **Primers** | **Amplicon Size** | **Forward (5’ – 3’)** | **Reference** |
| --- | --- | --- | --- | --- |
| mtCox1 | LCO | 689 bp | GGT CAA CAA ATC ATA AAG ATA TTG G | Folmer et al., 1994 |
|  | HCO |  | TAA ACT TCA GGG TGA CCA AAA AAT CA | Folmer et al., 1994 |
|  | polyLCO | 658 bp | GAY TAT WTT CAA CAA ATC ATA AAG ATA TTG G | Carr et al. 2011 |
|  | polyHCO |  | TAM ACT TCW GGG TGA CCA AAR AAT CA | Carr et al. 2011 |
| mt16S | 16sar | 524 bp | CGC CTG TTT ATC AAA AAA CAT | Palumbi, 1996 |
|  | 16sbr |  | ACG TGA TCT GAG TTC AGA CCG G | Palumbi, 1996 |

Carr, C. M., Hardy, S. M., Brown, T. M., Macdonald, T. A., Hebert, P. D. N. (2011). A tri-oceanic perspective: DNA barcoding reveals geographic structure and cryptic diversity in Canadian polychaetes. PLoS ONE, 6(7), e22232.

Folmer, O., Hoeh, W. R., Black, M. B., Vrijenhoek, R. C. (1994). Conserved primers for PCR

amplification of mitochondrial DNA from different invertebrate phyla. Molecular

Marine Biology and Biotechnology, 3(5), 294-299.

Palumbi, S. R. 1996. Nucleic acids II: The polymerase chain reaction. Pp. 205–248 in D. M. Hillis, C. Mortiz, and B. K. Mable, eds. Molecular systematics. Sinauer Associates, Sunderland, Mass.

**Table A2:** Summary of individual sequences and geographic locations for non- *Polyeunoa laevis* species and *P. laevis* separately. * indicates sequences were included in the *P. laevis* haplotype network but were not used in the Cytochrome c oxidase subunit I (Cox1) phylogenetic tree as they did not add any lineages.

| **Geographic sector** | **Location** | **Number of sequences** | **Source** | ***P. laevis* sequences** | ***P. laevis* source** |
| --- | --- | --- | --- | --- | --- |
| Antarctic continent | Amundsen Sea | 12 | Brasier et al. 2017 | 43* | Bogantes et al. 2020 |
|  | Adelie Land | 241 | This study | 14 | This study |
|  | Peninsula | 88 | This study | 10  45* | This study  Bogantes et al. 2020 |
|  | Ross Sea | 113 | This study | 98  21*  2 | This study  Bogantes et al. 2020  Gallego et al., 2013 |
| Sub-Antarctic | Kerguelen Archipelago | 72 | This study | 25 | This study |
| South America | Argentinean seamounts | 0 | - | 5* | Bogantes et al. 2020 |
|  | Tierra del Fuego | 16 | This study | 0 | - |
| Indian Ocean | Indian Ocean | 0 | - | 4 | Serpetti et al. 2017 |
| New Zealand | New Zealand Macquarie Ridge | 0 | - | 7* | Schiaparelli unpublished |
| Totals |  | 542 |  | 274 |  |

**Table A3**: List of polynoid samples collected from the Southern Ocean for this study. Species names are followed by the geographic location (Antarctic Peninsula, Ross Sea, Adélie Land, Kerguelen Archipelago, South America, and New Zealand), number of individuals, coordinates and GenBank accession numbers for the Cytochrome c oxidase subunit I (Cox1) sequences.

| **Species** | **Geographic Location** | **Number of individuals** | **Coordinates**  **(latitude longitude)** | **Cox1 accessions** |
| --- | --- | --- | --- | --- |
| *Austrolaenilla antarctica* | Antarctic Peninsula | 2 | -62° 55' 59.6634" -58° 40' 40.0074" | MT139454, MT139455 |
| *Austrolaenilla antarctica* | Ross Sea | 1 | -74° 40' 51.2394" 164° 12' 51.5988" | MT139456 |
| *Antarctinoe ferox* | Antarctic Peninsula | 5 | -63° 0' 31.4274" -58° 35' 35.5554"  -62° 44' 38.184" -57° 26' 39.696"  -62° 48' 1.188" -57° 4' 53.0034" | MT138961 - MT138965 |
| *Austrolaenilla pelagica* | Ross Sea | 1 | -74° 42' 9.4176" 164° 12' 18.0606" | MT139457 |
| *Barrukia cristata* | Adélie Land | 43 | -66° 39' 43.2" 140° 1' 51.6" -66° 39' 39.6" 140° 0' 28.8" | MT138993 - MT138996 MT138998 - MT139003 MT139005 - MT139027 MT139029 - MT139037 MT139062 |
|  | Antarctic Peninsula | 5 | -62° 55' 59.6634" -58° 40' 40.0074"  -62° 48' 1.188" -57° 4' 53.0034"  -63° 58' 48.792" -56° 46' 13.7994"  -63° 58' 46.7976" -56° 46' 14.4012" | MT139038 - MT139042 |
|  | Ross Sea | 19 | -74° 41' 12.3576" 164° 7' 22.0182"  -74° 41' 16.2594" 164° 8' 32.8194"  -74° 41' 41.2188" 164° 11' 4.4988"  -74° 41' 42.2982" 164° 4' 52.9212"  -74° 41' 50.3376" 164° 3' 32.9394"  -74° 41' 54.5382" 164° 7' 41.2212"  -74° 41' 8.217" 164° 7' 55.4412"  -74° 42' 2.9376" 164° 8' 15.4212"  -74° 42' 3.1176" 164° 8' 52.5582"  -74° 42' 48.117" 164° 8' 56.5188"  -74° 44' 12.3" 164° 10' 37.2612"  -74° 45' 43.4376" 164° 5' 46.4388"  -74° 46' 28.398" 164° 3' 39.2004" | MT139043 - MT139061 |
| *Barrukia* sp. | Adélie Land | 3 | -66° 39' 43.2" 140° 1' 51.6" | MT138997, MT139004, MT139028 |
| *Eunoe brunnea* | Adélie Land | 2 | -66° 39' 39.6" 140° 0' 28.8"  -66° 39' 43.2" 140° 1' 51.6" | MT139306, MT139307 |
|  | Antarctic Peninsula | 9 | -63° 51' 13.392" -55° 40' 55.8834"  -63° 0' 31.4274" -58° 35' 35.5554"  -62° 48' 1.188" -57° 4' 53.0034"  -62° 57' 9.8634" -58° 14' 20.364"  -62° 33' 47.7354" -56° 27' 18.5754"  -62° 44' 38.184" -57° 26' 39.696" | MT139308 - MT139316 |
| *Eulagisca gigantea* | Adélie Land | 1 | -66° 39' 43.2" 140° 1' 51.6" | MT139434 |
|  | Antarctic Peninsula | 1 | -62° 16' 2.316" -61° 11' 9.06" | MT139435 |

| *Eulagisca macnabi* | Antarctic Peninsula | 2 | -62° 48' 1.188" -57° 4' 53.0034" | MT139432, MT139433 |
| --- | --- | --- | --- | --- |
|  | Ross Sea | 1 | -74° 42' 9.4176" 164° 12' 18.0606" | MT139431 |
| *Eunoe opalina* | Antarctic Peninsula | 9 | -63° 0' 31.7988" -58° 35' 40.1994"  -62° 25' 56.9994" -56° 17' 15.6006" | MT138966 - MT138974 |
|  | Ross Sea | 1 | -74° 40' 51.2394" 164° 12' 51.5988" | MT138975 |
| *Eunoe* sp. | Kerguelen Archipelago | 2 | -48° 48' 24.8502" 70° 9' 44.496"  -49° 19' 18.9834" 69° 31' 49.0794" | MT138991, MT139452 |
|  | Antarctic Peninsula | 1 | -62° 48' 1.188" -57° 4' 53.0034" | MT139412 |
|  | South America | 15 | -53° 47' 13.1994" -70° 58' 26.4" | MT138976 - MT138990 |
| *Eulagisca* aff. *uschakovi* | Adélie Land | 3 | -66° 39' 43.2" 140° 1' 51.6" | MT139436 - MT139438 |
|  | Ross Sea | 1 | -74° 41' 41.2188" 164° 11' 4.4988" | MT139439 |
| *Eulagisca uschakovi* | Adélie Land | 9 | -66° 39' 43.2" 140° 1' 51.6"  -66° 39' 32.4" 140° 1' 26.4" | MT139440 - MT139442, MT139445 - MT139450 |
|  | Antarctic Peninsula | 3 | -63° 52' 0.588" -55° 35' 8.988"  -62° 48' 1.188" -57° 4' 53.0034"  -62° 25' 56.9994" -56° 17' 15.6006" | MT139443, MT139444, MT139451 |
| *Gorekia crassicirris* | Adélie Land | 4 | -66° 39' 43.2" 140° 1' 51.6" | MT138932 - MT138935 |
|  | Antarctic Peninsula | 6 | -63° 50' 20.4" -55° 37' 26.4"  -63° 1' 44.4" -58° 3' 17.99"  -62° 56' 6" -57° 58' 8.4"  -63° 0' 32.4" -58° 35' 41.99"  -62° 55' 49.8" -58° 41' 5.3982"  -63° 0' 31.7988" -58° 35' 40.1994" | MT138936 - MT138939  MT138956, MT138957 |
|  | Ross Sea | 16 | -74° 41' 52.799" 164° 7' 40.799"  -74° 45' 43.2" 164° 5' 45.6"  -74° 42' 3.5994" 164° 8' 52.7994" | MT138940 - MT138955 |
| *Gorekia* sp. | Antarctic Peninsula | 3 | -62° 56' 4.1994" -57° 58' 8.4"  -62° 56' 43.1982" -58° 23' 37.7988"  -58° 41' 5.3982" -62° 55' 49.8" | MT138958 - MT138960 |
| *Halosydna fuscomarmorata* | South America | 1 | -53° 47' 13.1994" -70° 58' 26.4" | MT138992 |
| *Harmothoe acuminata* | Antarctic Peninsula | 4 | -62° 44' 38.184" -57° 26' 39.696"  -62° 55' 59.6634" -58° 40' 40.0074" | MT139458 - MT139461 |
| *Harmothoe antarctica* | Adélie Land | 1 | -66° 39' 39.6" 140° 0' 28.8" | MT139400 |
|  | Antarctic Peninsula | 1 | -62° 55' 59.6634" -58° 40' 40.0074" | MT139401 |
| *Harmothoe crosetensis* | Adélie Land | 44 | -66° 39' 39.6" 140° 0' 28.8"  -66° 39' 43.2" 140° 1' 51.6" | MT139063 - MT139067  MT139069 - MT139070  MT139073 - MT139108  MT139110 |
|  | Antarctic Peninsula | 5 | -62° 48' 1.188" -57° 4' 53.0034"  -67° 33' 58.6794" -68° 6' 55.4394"  -64° 51' 10.7994" -62° 58' 37.1994"  -63° 58' 46.7976" -56° 46' 14.4012" | MT139068, MT139071, MT139072, MT139109, MT139136 |
|  | Ross Sea | 27 | -74° 41' 51.6582" 164° 7' 51.8982"  -74° 41' 12.3576" 164° 7' 22.0182"  -74° 41' 16.2594" 164° 8' 32.8194"  -74° 41' 24.957" 164° 6' 9.18"  -74° 41' 41.2188" 164° 11' 4.4988"  -74° 41' 42.2982" 164° 4' 52.9212"  -74° 41' 50.3376" 164° 3' 32.9394"  -74° 41' 8.217" 164° 7' 55.4412"  -74° 42' 0.36" 164° 2' 6.0612"  -74° 42' 17.9382" 164° 10' 15.9594"  -74° 42' 26.9994" 164° 10' 54.0012"  -74° 42' 48.117" 164° 8' 56.5188"  -74° 45' 43.4376" 164° 5' 46.4388"  -74° 46' 38.5176" 164° 3' 10.26" | MT139111 - MT139135, MT139137, MT139138 |
| *Harmothoe fuligineum* | Adélie Land | 106 | -66° 39' 39.6" 140° 0' 28.8"  -66° 39' 43.2" 140° 1' 51.6" | MT139139 - MT139149  MT139167 - MT139261 |
|  | Kerguelen Archipelago | 1 | -48° 40' 46.7646" 70° 58' 58.6632" | MT139150 |
|  | Antarctic Peninsula | 19 | -62° 44' 38.184" -57° 26' 39.696"  -62° 43' 47.9994" -57° 29' 24"  -62° 44' 38.184" -57° 26' 39.696"  -62° 57' 9.8634" -58° 14' 20.364"  -63° 51' 13.392" -55° 40' 55.8834"  -63° 51' 20.397" -55° 41' 6.6006"  -63° 58' 46.7976" -56° 46' 14.4012"  -63° 58' 48.792" -56° 46' 13.7994"  -64° 51' 10.7994" -62° 58' 37.1994"  -67° 33' 58.6794" -68° 6' 55.4394" | MT139151 - MT139166, MT139262, MT139283, MT139284 |
|  | Ross Sea | 38 | -74° 41' 12.3576" 164° 7' 22.0182"  -74° 41' 16.2594" 164° 8' 32.8194"  -74° 41' 24.957" 164° 6' 9.18"  -74° 41' 38.598" 164° 1' 51.8016"  -74° 41' 41.2188" 164° 11' 4.4988"  -74° 41' 42.2982" 164° 4' 52.9212"  -74° 41' 52.3314" 164° 8' 17.7288''  -74° 41' 54.5382" 164° 7' 41.2212"  -74° 41' 8.217" 164° 7' 55.4412"  -74° 41' 9.657" 164° 6' 56.7612"  -74° 42' 26.9994" 164° 10' 54.0012"  -74° 42' 3.1176" 164° 8' 52.5582"  -74° 42' 42.4182" 164° 9' 28.8612"  -74° 42' 48.117" 164° 8' 56.5188"  -74° 43' 22.1982" 164° 10' 23.3394"  -74° 43' 5.7972" 164° 14' 31.401"  -74° 46' 27.4794" 163° 57' 14.4"  -75° 8' 0.1782" 164° 31' 21.2412" | MT139263 - MT139282  MT139285 - MT139302 |
| *Harmothoe magellanica* | Kerguelen Archipelago | 33 | -48° 24' 32.832" 69° 1' 10.7394"  -48° 3' 25.3182" 70° 27' 9.3702"  -48° 33' 19.8354" 69° 14' 3.948"  -48° 38' 58.8546" 71° 5' 27.7614"  -48° 40' 7.4562" 70° 23' 59.9388"  -48° 5' 49.3254" 70° 33' 32.6406"  -48° 56' 18.6714" 70° 0' 21.1206"  -48° 57' 53.0712" 70° 43' 26.238"  -48° 59' 20.5614" 70° 45' 52.0806"  -49° 10' 25.8234" 69° 25' 36.012"  -49° 13' 41.142" 70° 59' 2.346"  -49° 13' 42.0852" 70° 43' 42.1068"  -49° 13' 48.936" 69° 35' 11.1474"  -49° 13' 57" 69° 33' 53.568"  -49° 18' 15.264" 70° 5' 15.288"  -49° 19' 18.9834" 69° 31' 49.0794"  -49° 21' 42.156" 69° 8' 51.756"  -49° 23' 15" 69° 56' 29.4"  -49° 23' 39.1194" 69° 56' 17.5194"  -49° 24' 21.3582" 70° 9' 31.6794"  -49° 25' 6.5922" 70° 51' 21.51"  -49° 38' 47.1654" 70° 38' 5.0382"  -49° 50' 12.2022" 69° 48' 29.7066"  -49° 56' 13.1208" 70° 0' 5.0682"  -50° 3' 50.6982" 69° 33' 11.1132" | MT139317 - MT139349 |
| *Harmothoe* sp*.* 1 | Kerguelen Archipelago | 11 | -48° 5' 49.3254" 70° 33' 32.6406"  -48° 5' 12.7386" 70° 16' 58.998"  -50° 17' 4.4586" 69° 42' 59.0394"  -48° 5' 12.7386" 70° 16' 58.998"  -48° 56' 18.6714" 70° 0' 21.1206" | MT139305, MT139402 - MT139411 |
| *Harmothoe* sp. 2 | Adélie Land | 11 | -66° 39' 43.2" 140° 1' 51.6"  -66° 39' 39.6" 140° 0' 28.8"  -66° 39' 43.2" 140° 1' 51.6" | MT139413 - MT139422, MT139453 |
|  | Antarctic Peninsula | 3 | -63° 58' 46.7976" -56° 46' 14.4012"  -64° 51' 10.7994" -62° 58' 37.1994"  -67° 33' 58.6794" -68° 6' 55.4394" | MT139424, MT139429, MT139430 |
|  | Ross Sea | 5 | -74° 40' 51.2394" 164° 12' 51.5988"  -74° 41' 24.957" 164° 6' 9.18"  -74° 41' 47.2776" 164° 7' 12.2412"  -74° 42' 48.117" 164° 8' 56.5188" | MT139423, MT139425 - MT139428 |
| *Polyeunoa laevis* Cluster 1 | Ross Sea | 44 | -74° 41' 50.3376" 164° 3' 32.9394"  -74° 40' 51.2394" 164° 12' 51.5988"  -73° 7' 28.2'' 174° 19' 13.8''  -74° 6' 40.2'' 170° 47' 45.6''  -74° 34' 54'' 170° 14' 59.4''  -74° 35' 24'' 170° 16' 33.6''  -74° 35' 25.8'' 170° 16' 32.4001''  -74° 40' 51.24'' 164° 12' 51.6''  -74° 41' 50.34'' 164° 3' 32.94''  -74° 41' 54.72'' 164° 7' 57.84''  -74° 41' 48.36'' 164° 7' 51.9''  -74° 44' 12.12'' 167° 3' 39.6''  -74° 44' 12.012'' 167° 3' 40.68''  -76° 35' 37.2'' 176° 49' 39''  -76° 46' 30'' 167° 50' 9.6'' | MT139303, MT139304, GU806038, GU806040, GU806041, GU806043, GU806045, HQ969766, HQ969768 - HQ969773, HQ969775, HQ969776, HQ969778, HQ969779, HQ969783, HQ969784, HQ969787 - HQ969795, HQ969797- HQ969802, HQ969804, HQ969808 - HQ969810, HQ969812, HQ969814 - HQ969817 |
|  | Adélie Land | 19 | -62° 44' 38.184" -57° 26' 39.696"  -62° 57' 9.8634" -58° 14' 20.364"  -66° 39' 43.2" 140° 1' 51.6"  -74° 41' 50.3376" 164° 3' 32.9394" | MT139381, MT139383, MT139385 - MT139387, MT139389, MT139392 - MT139399, MT139382, MT139384, MT139388, MT139391, MT139390 |
| *Polyeunoa laevis* Cluster 2 | Kerguelen Archipelago | 25 | -47° 39' 25.0308" 71° 51' 16.6428"  -47° 52' 32.9586" 71° 35' 16.2846"  -47° 53' 37.5174" 71° 43' 53.796"  -47° 9' 0.8568" 68° 31' 19.0482"  -48° 3' 25.3182" 70° 27' 9.3702"  -48° 5' 12.7386" 70° 16' 58.998"  -48° 6' 49.0314" 66° 12' 9.2262"  -49° 25' 6.5922" 70° 51' 21.51"  -49° 44' 17.1342" 65° 2' 49.0806"  -49° 46' 10.704" 67° 47' 2.6154"  -50° 0' 11.4906" 65° 35' 18.168"  -50° 11' 54.168" 64° 56' 37.0788"  -50° 2' 16.5042" 64° 56' 50.7474"  -50° 39' 22.2186" 69° 39' 25.5414"  -50° 6' 3.3408" 64° 48' 23.472" | MT139350 - MT139374 |
| *Polyeunoa laevis* Cluster 3 | Antarctic Peninsula | 6 | -62° 44' 24" -57° 26' 23.9994"  -62° 44' 38.184" -57° 26' 39.696"  -62° 57' 9.8634" -58° 14' 20.364" | MT139375 - MT139380 |
|  | Ross Sea | 49 | -67° 50' 13.8001'' -179° 33' 37.1999''  -69° 25' 54.5999'' -178° 49' 10.2''  -68° 5' 33.6'' -179° 15' 42''  -68° 6' 38.4001'' -179° 18' 56.9999''  -72° 35' 25.08'' 175° 20' 32.28''  -67° 22' 55.2'' -179° 52' 19.2''  -67° 51' 15.5999'' -179° 38' 40.2''  -67° 49' 43.8'' -179° 35' 13.2''  -68° 6' 31.7999'' -179° 14' 17.9999''  -67° 22' 60'' -179° 50' 39.6''  -72° 4' 31.8'' 172° 54' 15.5999''  -67° 21' 0.5987'' 179° 52' 40.8216''  -71° 56' 18.6'' 173° 18' 8.28''  -68° 5' 19.2'' -179° 17' 55.2''  -74° 34' 54'' 170° 14' 59.4''  -67° 22' 14.4'' 179° 58' 49.8''  -67° 24' 29.4001'' -179° 48' 33.0001''  -73° 7' 28.2'' 174° 19' 13.8'' | GU806017, GU806018, GU806021, GU806023, GU806024, GU806026, GU806030, GU806031, GU806035 - GU806037, GU806044, GU806047, GU806050, GU806051, GU806055, GU806056, GU806060, GU806061, GU806063, GU806065, GU806066, GU806070, HQ969755 - HQ969758, HQ969760 - HQ969765, HQ969767, HQ969774, HQ969777, HQ969822, HQ969823, HQ969780 - HQ969782, HQ969786, HQ969796, HQ969803, HQ969805, HQ969806, HQ969807, HQ969811, HQ969813 |
| *Polyeunoa laevis* Cluster 4 | New Zealand | 7 | -42° 39' 16.812'' -177° 12' 47.52''  -42° 38' 39.5999'' -179° 52' 52.7999'' | HQ578304 - HQ578308  HQ969829, HQ969830 |
| *Parapolyeunoa flynni* | Ross Sea | 1 | -71° 51' 20.4'' 174° 1' 58.8'' | GU806049 |

**Summary A1**

Samples were retrieved from the areas of interest during the following collection campaigns:

PNRA XXIX Exp 13/14 (Ross Sea)

PNRA XXV Exp 09/10 (Ross Sea)

PNRA XXVII Exp 11/12 (Ross Sea)

PNRA XXVII Exp 12/13 (Ross Sea)

CEAMARC (Adélie Land)

POLARIS (Adélie Land)

POLARIS 2015 (Adélie Land)

POLARIS 2016 (Adélie Land)

REVOLTA (Adélie Land)

POKER II (Kerguelen Archipelago)

PROTEKER (Kerguelen Archipelago)

ANT XXIX-3 (Antarctic peninsula)

Polarstern PS81 XXIX/3 (Antarctic peninsula)

IPY-CAML TAN0802 (Antarctica)

(New Zealand) (NIWA collection numbers: 28791a, 28791b, 28791c, 28791d, 28791e, 53275a, 53275b)

Further, samples were retrieved from the areas of interest thanks to the following individuals: Melody Clark (Antarctic peninsula), Marie-Laure Guillemain (Antarctic peninsula), Karin Gerard (South American peninsula), Cyril Gallut (Adélie Land), Melyne Hautecoeur (Antarctic peninsula) andMarc Eléaume (Antarctic peninsula).

Additional notes:

For the PNRA expedition, most of the samples obtained were collected via trawling or hand-picked during diving. The sampling year is denoted in the expedition names (e.g., 13/14 indicates 2013 to 2014). PNRA website: <https://www.italiantartide.it/>.

For the AWI expedition, all samples were collected using an Agassiz trawl during 2013. The expedition report is available here: <https://www.tib.eu/en/search?tx_tibsearch_search%5Baction%5D=download&tx_tibsearch_search%5Bcontroller%5D=Download&tx_tibsearch_search%5Bdocid%5D=awi%3Adoi~10.2312%252FBzPM_0672_2014&cHash=957e893e7b7d84d93814e12b88b4cc95#download-mark>
